# Random coil shifts of posttranslationally modified amino acids

**DOI:** 10.1007/s10858-019-00270-4

**Published:** 2019-07-17

**Authors:** Anne C. Conibear, K. Johan Rosengren, Christian F. W. Becker, Hanspeter Kaehlig

**Affiliations:** 1 Faculty of Chemistry, Institute of Biological Chemistry, University of Vienna, Währinger Straße 38, 1090 Vienna, Austria; 2 School of Biomedical Sciences, The University of Queensland, QLD 4072 Brisbane, Australia; 3 Faculty of Chemistry, Institute of Organic Chemistry, University of Vienna, Währinger Straße 38, 1090 Vienna, Austria

**Keywords:** Nuclear magnetic resonance spectroscopy, Peptides, Posttranslational modification, Protein modification, Random coil shifts, Secondary structure

## Abstract

Most eukaryotic proteins are modified during and/or after translation, regulating their structure, function and localisation. The role of posttranslational modifications (PTMs) in both normal cellular processes and in diseases is already well recognised and methods for detection of PTMs and generation of specifically modified proteins have developed rapidly over the last decade. However, structural consequences of PTMs and their specific effects on protein dynamics and function are not well understood. Furthermore, while random coil NMR chemical shifts of the 20 standard amino acids are available and widely used for residue assignment, dihedral angle predictions and identification of structural elements or propensity, they are not available for most posttranslationally modified amino acids. Here, we synthesised a set of random coil peptides containing common naturally occurring PTMs and determined their random coil NMR chemical shifts under standardised conditions. We highlight unique NMR signatures of posttranslationally modified residues and their effects on neighbouring residues. This comprehensive dataset complements established random coil shift datasets of the 20 standard amino acids and will facilitate identification and assignment of posttranslationally modified residues. The random coil shifts will also aid in determination of secondary structure elements and prediction of structural parameters of proteins and peptides containing PTMs.

**Electronic supplementary material:**

The online version of this article (10.1007/s10858-019-00270-4) contains supplementary material, which is available to authorized users.

## Introduction

Posttranslational modifications (PTMs) expand the complexity of the proteome and their role in altering protein function and regulation is well recognised (Walsh et al. 2005; Aebersold et al. 2018). Addition of small chemical functionalities (e.g. phosphate-, acetyl-, methyl-groups etc.), or more complex molecules such as carbohydrates or other polypeptides to proteins, in a controlled fashion, regulates fundamental cellular processes like transcription, cellular signalling and protein homeostasis. For example, a delicate balance between PTM addition and removal at specific locations and in particular combinations on histone proteins regulates the compactness of chromatin and thereby the rate of transcription (Muller and Muir 2015). Furthermore, errors in addition, removal or location of PTMs can lead to protein dysregulation and are increasingly associated with disease processes. For example, modification of the Parkinson’s disease-associated protein α-synuclein with the carbohydrate *O*-GlcNAc tends to inhibit the formation of toxic aggregates (Lewis et al. 2017). The Alzheimer’s disease-associated protein Tau-4 is also known to be extensively modified and the cross-talk between phosphorylation and glycosylation modifications has been studied using NMR spectroscopy (Bourre et al. 2018). Furthermore, incorporation of carboxymethyllysine in the central repeat region increased aggregation of Tau-4 but incorporation of phosphoserine decreased aggregation (Ellmer et al. 2019). These, amongst many other studies, illustrate the importance of understanding the role of PTMs in protein structure and function.

The role of PTMs is largely underrepresented in structural biology, although protein NMR spectroscopy is well suited to studying structural and dynamic properties of proteins in near-native conditions (Theillet et al. 2012a). Our understanding of the prevalence and diversity of PTMs has grown with developments in mass spectrometry and antibody-based techniques for detecting and locating PTMs on proteins and tens of thousands of proteins in the SwissProt database are annotated with PTMs (Aebersold et al. 2018). NMR spectroscopy studies, however, are predominantly carried out using recombinant proteins that lack the relevant PTMs. Access to posttranslationally modified proteins is now facilitated by recent developments in protein chemical synthesis (Conibear et al. 2018), genetic code engineering (Liu and Schultz 2010; Lang and Chin 2014) and enzymatic protein modification (Zhang et al. 2018), enabling studies of the role of PTMs in protein structure and function. Furthermore, increased commercial availability of the requisite building blocks for solid phase peptide synthesis (SPPS) means that peptides and proteins bearing PTMs are finding application in the pharmaceutical industry, chemical biology and peptide-based materials (Barber and Rinehart 2018).

Random coil chemical shifts are widely used in NMR structural biology of proteins and peptides but have only been determined for the 20 standard amino acids (Wishart et al. 1995; Schwarzinger et al. 2000), with the exception of three phosphorylated residues (Bienkiewicz and Lumb 1999). Several sets of random coil NMR chemical shifts obtained under a number of conditions are available, and readers are directed to two extensive reviews for a comparison of the datasets, advice on choosing an appropriate set of values and their various applications (Wishart and Nip 1998; Mielke and Krishnan 2009). The majority of random coil shifts has been determined using glycine or alanine as flanking residues but glutamine has also been suggested as a flanking residue that might provide better predictions of Ramachandran distribution (Ting et al. 2010; Kjaergaard and Poulsen 2011). In addition to aiding spectral assignment, comparison of measured chemical shifts with random coil shifts can give an indication of secondary structural elements, hydrogen bonding, oxidation states, protonation states and structural propensity of disordered regions (Mielke and Krishnan 2004, 2009). Recently, characteristic chemical shift patterns have been used to determine NMR sequence tags that can be used to sequence peptides or small proteins such as conotoxins by comparison with a sequence database (Wilson and Daly 2018).

Much work has been directed towards deriving structural restraints from chemical shifts for use in protein structure calculations and being able to predict protein structure from chemical shift data. Most of these approaches use secondary chemical shifts (the difference between an observed chemical shift and the respective random coil value) as the initial input data. The chemical shift index (CSI) has been widely used to predict the type and location of secondary structural elements in proteins from ^1^H chemical shifts (Wishart et al. 1992); although not strictly secondary shifts, the deviation of the Hα shifts from a table of mid-point reference Hα shift ranges is calculated for each residue and used to allocate it a score (Wishart et al. 1992). The widely-used programme TALOS-N (Shen and Bax 2013), and its predecessors TALOS + (Shen et al. 2009) and TALOS (Cornilescu et al. 1999), predicts backbone φ and ψ angles, as well as some side-chain χ_1_ angles, by matching secondary chemical shifts of peptide segments to those in a database. Furthermore, an *S*^2^ model-free order parameter is predicted that can indicate the flexibility of a protein region (Berjanskii and Wishart 2005). These predictions are used to validate structures derived from NOE correlations or are added as additional restraints in protein structure calculations. Another example of a prediction tool that uses secondary chemical shift input data is ‘DISH’, a method for predicting the dihedral angles of cystine side chains and disulfide bond conformations (Armstrong et al. 2018). Finally methods such as CS-ROSETTA (Shen et al. 2008, 2009) utilise chemical shifts for de novo structure determination of proteins, and more specialised approaches focussing on disulfide-rich peptides have also been described recently (Wang and Craik 2019). For proteins containing PTMs, the lack of random coil reference values of posttranslationally modified residues means that predictions for some residues cannot be made, or are compromised by using the random coil shift of the corresponding unmodified residue. This affects both the modified residue and its neighbours, and can prevent, or lead to inaccurate, structural predictions for these residues.

Random coil shifts of posttranslationally modified residues have not been systematically characterised, although several studies have highlighted distinctive NMR features of PTMs and random coil shifts of phosphorylated serine, threonine and tyrosine have been determined (Bienkiewicz and Lumb 1999). A dataset of observed ^1^H and ^13^C chemical shifts of posttranslationally modified residues in conotoxins was compiled and analysed by Marx et al. (2006), providing a useful reference for studies of conotoxins. This dataset, however, was derived from folded peptides with defined structures and so does not have the same broad applications as random coil chemical shifts. A novel method for identification of PTMs, especially glycosylation, on unlabelled protein samples was introduced by Schubert et al., involving denaturation of glycosylated proteins and identification of the distinct NMR fingerprint patterns of the glycosyl moieties (Schubert et al. 2015). NMR signatures of N-terminal gluconoylation have also been reported and should be useful for identifying this PTM, which is common in recombinantly expressed His-tagged proteins (Schweida et al. 2019). The value of NMR spectroscopy for monitoring protein modification and associated changes in protein structure has been illustrated in several studies by Selenko and co-workers (Dose et al. 2011; Liokatis et al. 2010; Theillet et al. 2012, 2013). For example, they showed that lysine CH_2_ε resonances can be used to distinguish acetylation and methylation events and were able to monitor the kinetics of the modification in the complex environment of cell extracts (Theillet et al. 2012b).

Here we present a set of random coil NMR chemical shifts of common posttranslationally modified residues for use in assignment and secondary structure determination of modified proteins and peptides and identification of protein PTMs. We selected the modified residues based on their prevalence in eukaryotic proteins, their commercial availability, and their stability and ease of incorporation into proteins and peptides by SPPS. The set of random coil peptides was synthesised by SPPS and the ^1^H, ^13^C and ^15^N chemical shifts were determined in 9:1 H_2_O/D_2_O at pH 5. The pH dependence of chemical shifts in γ-carboxyglutamate and carboxymethyl lysine was also determined. We present the random coil shifts and compare them with those of the corresponding unmodified residues, highlighting some distinctive characteristics of the modified residues. The random coil shifts of posttranslationally modified residues can be applied to any protein and used in combination with established random coil shifts of standard residues to facilitate our understanding of the role of PTMs in protein structure and function.

## Materials and methods

### Solid phase peptide synthesis of random coil peptides

The random coil peptides Ac–G–G–X–G–G–NH_2_, where ‘X’ is the modified or unmodified residue of interest, were synthesised either manually or on an automated microwave peptide synthesiser (Liberty Blue, CEM, methods in Supplementary Information Table S1) on Rink amide resin at 0.05 mmol scale according to the following procedure. Special procedures for particular modified residues or protecting groups are detailed in Table S1. 9-fluorenylmethoxycarbonyl (Fmoc)-protected building blocks (listed in Table S1, 2.5 eq.) were manually coupled using 2-(1*H*-benzotriazol-1-yl)-1,1,3,3-tetramethyluronium hexafluorophosphate (HBTU, 2.4 eq., 0.5 M in DMF) as activator in combination with diisopropylethylamine (DIPEA, 5 eq.) as base under rotation at room temperature for 20–30 min. N-terminal deprotection was achieved with piperidine [20% in dimethylformamide (DMF), 2 × 5 min]. After removal of the Fmoc protecting group, the final N-terminal glycine was acetylated with acetic anhydride/DCM/DIPEA 10:85:5 (2 × 5 min). The resin was dried and the peptide cleaved from the resin with trifluoroacetic acid (TFA)/triisopropylsilane(TIPS)/H_2_O 95:2.5:2.5 for 2 h and then precipitated with diethyl ether and pelleted by centrifugation. The crude peptide was dissolved in acetonitrile/water 1:1 and lyophilised 2–3 times to remove volatile components from the cleavage, before analysis by ESI–MS in positive ion mode. Specific methods for particular modified residues and supplier details are given in Table S1.

### NMR data collection and processing

Samples for NMR data collection were prepared by dissolving the crude lyophilised peptide (3–5 mg) in 600 μL H_2_O/D_2_O 9:1 containing 166 μM 2,2-dimethyl-2-silapentane-5-sulfonate sodium salt (DSS) as an internal reference (Wishart et al. 1995). All peptides were sufficiently pure to allow unambiguous peak assignments. The pH was carefully adjusted to pH 5.0 ± 0.3 with NaOH and HCl, using a calibrated pH meter with a microelectrode. Masses of crude peptide and pH of each sample are given in Table S2.

NMR spectra were acquired at the University of Vienna NMR Centre on an Avance III HDX 700 MHz NMR spectrometer (Bruker BioSpin, Germany) equipped with an inverse helium cooled quadruple cryoprobe (QCI-F) with a resonance frequency of 700.40 MHz for ^1^H, 176.12 MHz for ^13^C, and 70.97 MHz for ^15^N, respectively. Spectra were acquired at 298 K and standard Bruker pulse sequences were used with water suppression by either presaturation or 3-9-19 WATERGATE. Spectra acquired for each peptide included: ^1^H (zgpr); ^1^H–^15^N HSQC (hsqcetgpsi2); ^1^H–^1^H TOCSY (mlevgpph19, D9 isotropic mixing time = 100 ms); ^1^H–^1^H NOESY (noesygpph19, D8 mixing time = 800 ms); ^1^H–^13^C HSQC (hsqcetgp); ^1^H–^13^C HMBC (hmbcgplpndprqf). Sweep widths were generally ^1^H 11.0 ppm, ^13^C 60.0 ppm for HSQC, 180.0 ppm for HMBC, and ^15^N 36.0 ppm The total time to acquire all six spectra was approximately 15 h per peptide.

Spectra were Fourier transformed, phased and calibrated on the DSS signal (^1^H at 0 ppm) in Topspin (Bruker BioSpin, Germany). ^15^N and ^13^C spectra were calibrated on the unified scale according to the IUPAC recommendations (Wishart et al. 1995; Harris et al. 2008), using a ratio of 0.251449530 for ^13^C and 0.101329118 for ^15^N. Spectra were assigned in CCP-NMR (Vranken et al. 2005), independently of previously assigned random coil shifts. ^1^H shifts were assigned based on spin system identification using TOCSY spectra and dαN(*i*, *i *+ 1) connectivities were assigned using the sequential assignment protocol in the NOESY spectra (Wüthrich 1986). ^15^N and ^13^C chemical shifts were derived from HSQC and HMBC spectra.

### pH titrations

Samples of peptides containing γ-carboxyglutamic acid and carboxymethyl lysine as residue ‘X’ were prepared as above and the pH was adjusted to pH 2.0 with HCl. NMR spectra were acquired at the Centre for Advanced Imaging (The University of Queensland) on an Avance 700 MHz NMR spectrometer (Bruker BioSpin, Germany) with a cryoprobe. At pH 2.0 and at pH 9.0, ^1^H, ^1^H–^1^H TOCSY, ^1^H–^15^N HSQC, ^1^H–^13^C HSQC and ^1^H–^13^C HMBC spectra were acquired to enable assignment of the ^1^H, ^15^N and ^13^C resonances at the extreme pH values. The pH was then increased in ~ 0.5 steps using 0.1 M HCl and 0.1 M NaOH and ^1^H and ^1^H–^1^H TOCSY spectra were acquired at each step to provide ^1^H chemical shifts. Spectra were processed, referenced and assigned as above.

## Results and discussion

### Design and synthesis of random coil peptides

**Fig. 1:**
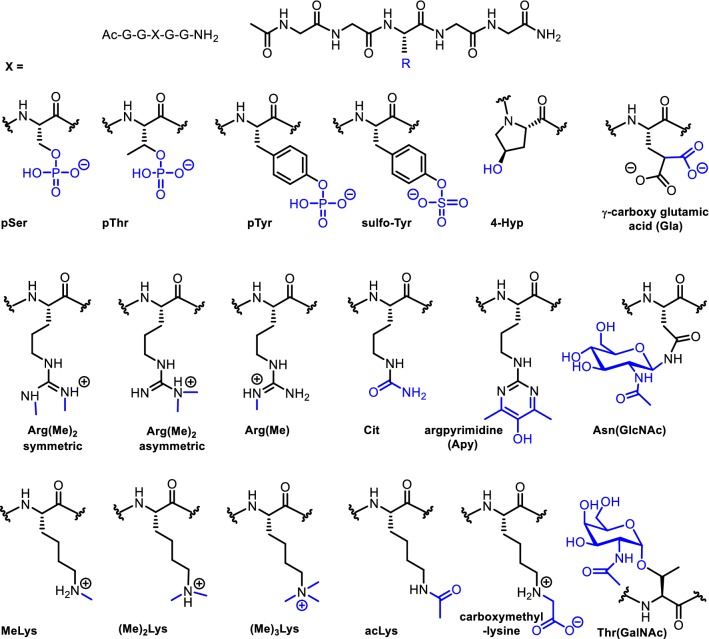
Random coil peptide design and structures of the modified amino acids included in this study. PTMs are highlighted in blue. Protonation states at pH 5.0 are estimated based on available pK_a_ values

The amino acids bearing PTMs (Fig. 1) were selected based on their relevance as naturally occurring protein PTMs, their stability and the availability of suitably protected building blocks for SPPS (Table S1). Random coil peptides were designed based on a commonly used peptide design (Fig. 1) (Schwarzinger et al. 2000; Bienkiewicz and Lumb 1999; Plaxco et al. 1997; Schwarzinger et al. 2001), comprising the modified residue flanked by two glycine residues and the N- and C-termini blocked as the acetyl and amide derivatives, respectively, to prevent interactions of the charged termini. The peptides were synthesised by manual or automated Fmoc-based SPPS and peptides containing the corresponding unmodified residues were synthesised as controls. Most of the modified amino acid building blocks can be easily incorporated using standard Fmoc SPPS protocols, with some requiring additional steps to remove protecting groups (Table S1). The set of random coil peptides was successfully synthesised and was characterised by mass spectrometry (structures and mass spectra are provided in the Supplementary Information). Due to the size and hydrophilicity of the peptides, purification by RP-HPLC was not possible and the crude peptides were deemed of sufficient purity (approximately 70–95% based on mass spectrometry and NMR data), for unambiguous assignment of the resonances. We note that other widely-used random coil shift datasets have also been determined using crude peptides, but analytical data were not published for comparison (Schwarzinger et al. 2000; Plaxco et al. 1997).

### Assignment of random coil chemical shifts

Samples for NMR spectroscopy (~ 15 mM) were prepared in H_2_O/D_2_O 9:1 at pH 5.0 ± 0.3 (amounts and pH for each peptide are shown in Table S2) and were referenced to internal DSS (Wishart et al. 1995). These conditions enable comparison with published random coil shifts of the standard amino acids and allow for assignment of ^1^H, ^13^C and ^15^N chemical shifts at natural abundance. ^1^H, TOCSY, NOESY, ^15^N-HSQC, ^13^C-HSQC and ^13^C-HMBC spectra were acquired at 298 K and chemical shifts were assigned using the sequential assignment protocol in CCP-NMR (Vranken et al. 2005; Wüthrich 1986). ^1^H spectra were acquired at the beginning and end of the set of experiments to ensure that the peptides and modifications were stable under the NMR conditions for the duration of the experiment (~ 15 h). Chemical shift assignments for the modified residues and their unmodified counterparts are given in Table 1. Chemical shifts of the flanking glycine residues are given in the Supplementary Information (Table S3).

**Table 1:** Random coil chemical shifts (ppm) of modified and unmodified residues ‘X’

Residue ‘X’	NH	HN	Hα	Hβ	Cα	Cβ	C=O	Others
Ser	115.9	8.40	4.50	3.88, 3.93	58.6	63.8	175.5	
pSer	115.9	8.72	4.58	4.12, 4.20	57.6	66.7	175.1	
Thr	113.1	8.22	4.39	4.31	62.0	69.8	175.7	Hγ 1.21, Cγ 21.5
pThr	113.6	8.47	4.43	4.65	61.9	73.9	175.2	Hγ 1.33, Cγ 20.8
Thr(GalNAc)	111.8	8.39	4.63	4.42	60.5	78.1	174.9	Hγ 1.26, Cγ 20.6; H1 4.93, C1 101.4; H2 4.09, C2 52.7; H3 3.90, C3 70.5; H4 3.97, C4 71.4; H5 4.02, C5 74.1; H_2_C6 3.74, 3.75, CH_2_6 64.1; Ac(NH 123.1, HN 7.92, CO 177.0, H_3_C 2.02, CH_3_ 25.0)
Tyr	120.5	8.21	4.57	2.98, 3.08	58.2	38.7	176.8	Cγ 130.7; Hδ 7.15, Cδ 133.3; Hε 6.85, Cε 118.3; Cζ 157.3
pTyr	120.2	8.24	4.61	3.02, 3.14	58.1	38.8	176.8	Cγ 134.4; Hδ 7.22, Cδ 133.0; Hε 7.14, Cε 123.3; Cζ 154.0
Tyr(SO_3_)	120.0	8.24	4.63	3.06, 3.18	57.9	38.8	176.6	Cγ 136.9; Hδ 7.28, Cδ 133.2; Hε 7.26, Cε 124.4; Cζ 152.9
Lys	120.9	8.31	4.35	1.78, 1.88	56.5	32.9	177.5	Hγ 1.41, 1.46, Cγ 24.7; Hδ 1.68, Cδ 29.0; Hε 3.00, Cε 42.2
Lys(ac)	121.4	8.28	4.31	1.74, 1.84	56.8	33.1	177.7	Hγ 1.34, 1.39, Cγ 25.1; Hδ 1.51, Cδ 30.5; Hε 3.16, Cε 42.0; Hζ 7.94, Nζ 127.3; Ac(CO 176.8; H_3_C 1.97, CH_3_ 24.6)
Lys(Me)	120.9	8.31	4.35	1.77, 1.88	56.4	32.9	177.4	Hγ 1.41, 1.45, Cγ 24.7; Hδ 1.69, Cδ 27.6; Hε 3.03, Cε 51.6; H_3_C 2.70, CH_3_ 35.6
Lys(Me)_2_	120.8	8.31	4.36	1.78, 1.89	56.4	32.9	177.4	Hγ 1.41, 1.44, Cγ 24.6; Hδ 1.73, Cδ 26.2; Hε 3.12, Cε 60.2; (H_3_C)_2_ 2.86, (CH_3_)_2_ 45.4
Lys(Me)_3_	120.7	8.31	4.37	1.80, 1.91	56.3	32.9	177.4	Hγ 1.40, 1.45, Cγ 24.6; Hδ 1.81, Cδ 24.5; Hε 3.31, Cε 68.9; (H_3_C)_3_ 3.10, (CH_3_)_3_ 55.5
Carboxymethyl lysine (CML)	121.0	8.31	4.35	1.78, 1.88	56.5	32.9	177.5	Hγ 1.42, 1.46, Cγ 24.8; Hδ 1.72, Cδ 27.8; Hε 3.06, Cε 50.0; H_2_C 3.60, CH_2_ 52.0; CO 174.1
Arg	120.7	8.34	4.37	1.78, 1.91	56.3	30.6	177.3	Hγ 1.62, 1.66, Cγ 27.1; Hδ 3.21, Cδ 43.3; HNε 7.20^a^; Cζ 159.6
Arg(Me)	120.7	8.33	4.36	1.78, 1.90	56.4	30.7	177.3	Hγ 1.62, 1.65, Cγ 27.1; Hδ 3.21, Cδ 43.3; HNε 6.97^a^; Cζ 159.3; HNη 6.85^a^; H_3_C 2.82, CH_3_ 30.2
Arg(Me)_2_ symmetric (SDMA)	120.7	8.33	4.36	1.78, 1.90	56.4	30.7	177.3	Hγ 1.62, 1.65, Cγ 27.1; Hδ 3.22, Cδ 43.1; HNε 6.77^a^, NHε 115.9; Cζ 158.7; HNη 6.78^a^; (H_3_C)_2_ 2.81, 2.82, (CH_3_)_2_ 30.1
Arg(Me)_2_ asymmetric (ADMA)	120.7	8.33	4.37	1.78, 1.90	56.4	30.7	177.3	Hγ 1.63, 1.66, Cγ 27.3; Hδ 3.26, Cδ 44.1; HNε 6.79^a^, NHε 119.5; Cζ 158.8; HNη 6.71^a^, NHη 107.9; (H_3_C)_2_ 3.00, (CH_3_)_2_ 40.3
Argpyrimidine (Apy)	121.1	8.31	4.37	1.80, 1.92	56.6	30.8	177.5	Hγ 1.64, 1.69, Cγ 27.5; Hδ 3.38, Cδ 43.5; Cζ 156.6; 3C 141.0; 4C 160.9; (H_3_C)_2_ 2.36, (CH_3_)_2_ 20.1
Cit	121.1	8.32	4.34	1.74, 1.86	56.6	30.8	177.6	Hγ 1.51, 1.56, Cγ 28.2; Hδ 3.11, Cδ 42.0; HNε 6.33^a^, NHε 123.7; Cζ 164.3; H_2_N 5.56^a^
Pro (*trans*)	–	–	4.45	1.99, 2.29	63.7	32.0	178.1	Hγ 2.04, Cγ 27.2; Hδ 3.64, 3.67, Cδ 49.8
Pro (*cis*, ~ 11.5%)^b^	–	–	4.63	2.17, 2.38	63.0	34.6	n.d.^c^	Hγ 1.87, 1.96, Cγ 24.7; Hδ 3.54, 3.61, Cδ 50.3
4-Hyp (*trans*)	–	–	4.56	2.12, 2.36	62.2	39.7	177.5	Hγ 4.64, Cγ 72.6; Hδ 3.65, 3.82, Cδ 57.3
4-Hyp (*cis *~ 9%)^b^	–	–	4.58	2.28, 2.52	62.2	42.3	n.d.^c^	Hγ 4.53, Cγ 70.6; Hδ 3.60, 3.76, Cδ 57.5
Asn	118.8	8.47	4.76	2.80, 2.86	53.3	38.9	176.3	Cγ 177.4; H_2_Nδ 6.91, 7.61, NH_2_δ 112.8
Asn(GlcNAc)	118.8	8.49	4.78	2.82, 2.85	52.9	39.3	176.1	Cγ 175.5; H_2_Nδ 8.67, NH_2_δ 131.8; H1 5.04, C1 81.1; H2 3.81, C2 57.1; H3 3.60, C3 77.1; H4 3.47, C4 72.3; H5 3.51, C5 80.4; H_2_C6 3.75, 3.88, CH_2_6 63.4; Ac(HN 8.18, NH 122.1, CO 177.6, H_3_C 2.00, CH_3_ 24.9)
Glu	120.7	8.50	4.34	1.98, 2.11	56.7	29.6	177.3	Hγ 2.34, Cγ 35.1; Cδ 182.8
γ-Carboxy glutamic acid (Gla)	120.2	8.56	4.37	2.20, 2.36	55.7	33.5	177.1	Hγ 3.27, Cγ 54.9; Cδ1/Cδ2 179.1
Gly	109.1	8.42	4.01	–	45.5	–	175.1	

^a^Line broadening observed indicating possible multiple conformations or solvent exchange

^b^The proportion of minor peptide conformations containing *cis*-proline or *cis*-hydroxyproline were estimated by integration of 3–4 well-resolved signals in the ^1^H spectra

^c^n.d. = not determined. Carbonyl resonances of the minor conformations could not be assigned unambiguously

The ^1^H–^1^H NOESY spectra of the random coil peptides showed only strong (*i*, *i *+ 1) inter-residue crosspeaks, indicating that the peptides are indeed ‘random coil’ and have no long-range interactions (Bienkiewicz and Lumb 1999; Dyson and Wright 1991). Furthermore, we compared the ^1^H and ^13^C spectra of four peptides (X = serine, phosphoserine, lysine and acetyllysine) acquired in 8 M urea, pH 2.3 with those acquired in water and found no major differences in NOESY crosspeaks or chemical shifts (Table S4, except for phosphoserine—as discussed below), confirming that the peptides are in an extended conformation. These validations of the random coil nature of the peptides used in this study are important for use of the random coil shifts as a standard reference dataset to which the observed chemical shifts of any protein can be compared to identify structural elements.

**Fig. 2:**
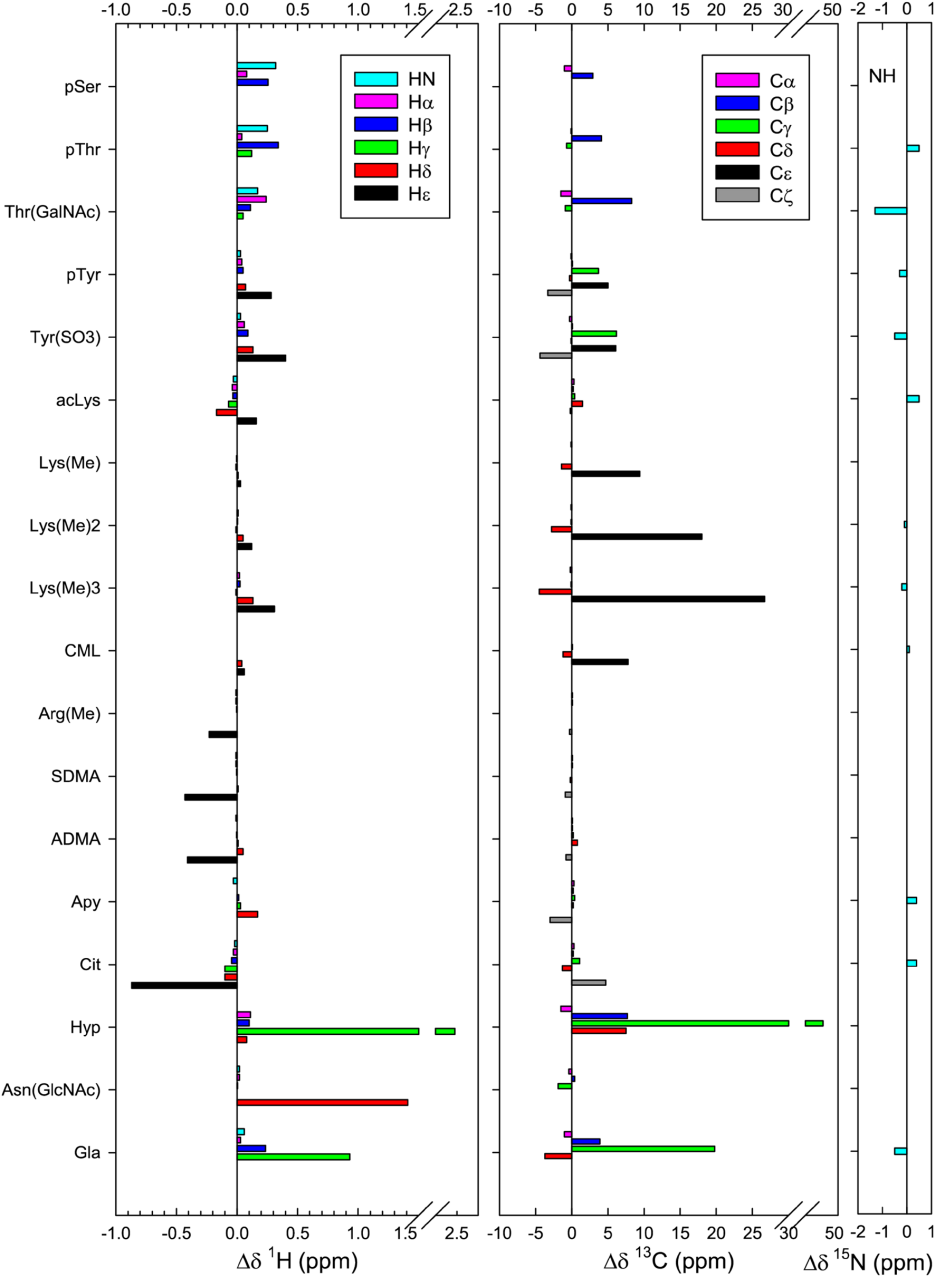
^1^H, ^13^C and ^15^N chemical shift differences (Δδ) between modified residues and their corresponding unmodified residues. CO differences are all ≤ 0.8 ppm and have been omitted for clarity

The assigned random coil shifts in Table 1 were compared with corresponding unmodified residues, the same PTM on different residues, and literature values. The chemical shifts of the unmodified residues are in good agreement with widely used random coil shifts in the literature (Table S5) (Wishart et al. 1995; Schwarzinger et al. 2000), showing that the set of random coil shifts of modified residues complements and can be used in conjunction with those already established. Comparison of the chemical shifts of modified and unmodified residues (Fig. 2) reveals that, for most of the random coil peptides, addition of a PTM made little or no difference to the backbone chemical shifts of the residue (NH, HN, Hα, Cα and CO). This is unsurprising as most PTMs occur at the termini of solvent-exposed side chains, where they are unlikely to alter the shielding of the backbone nuclei. However, modifications of serine and threonine are exceptions; HN shifts downfield by 0.32 ppm for serine and 0.25 ppm for threonine phosphorylation. Furthermore, O-linked glycosylation of threonine by N-acetylgalactosamine causes a 1.3 ppm upfield shift in NH and a 0.24 ppm downfield shift in Hα, which was not observed for N-linked glycosylation of asparagine by *N*-acetylglucosamine. These changes indicate (transient) hydrogen bond formation between the PTM and the backbone NH, as has been reported for serine phosphorylation (Du et al. 2005) and suggested for O-linked glycosylation, as discussed further below (Martinez-Saez et al. 2017).

### Phosphorylation of serine, threonine and tyrosine

**Fig. 3:**
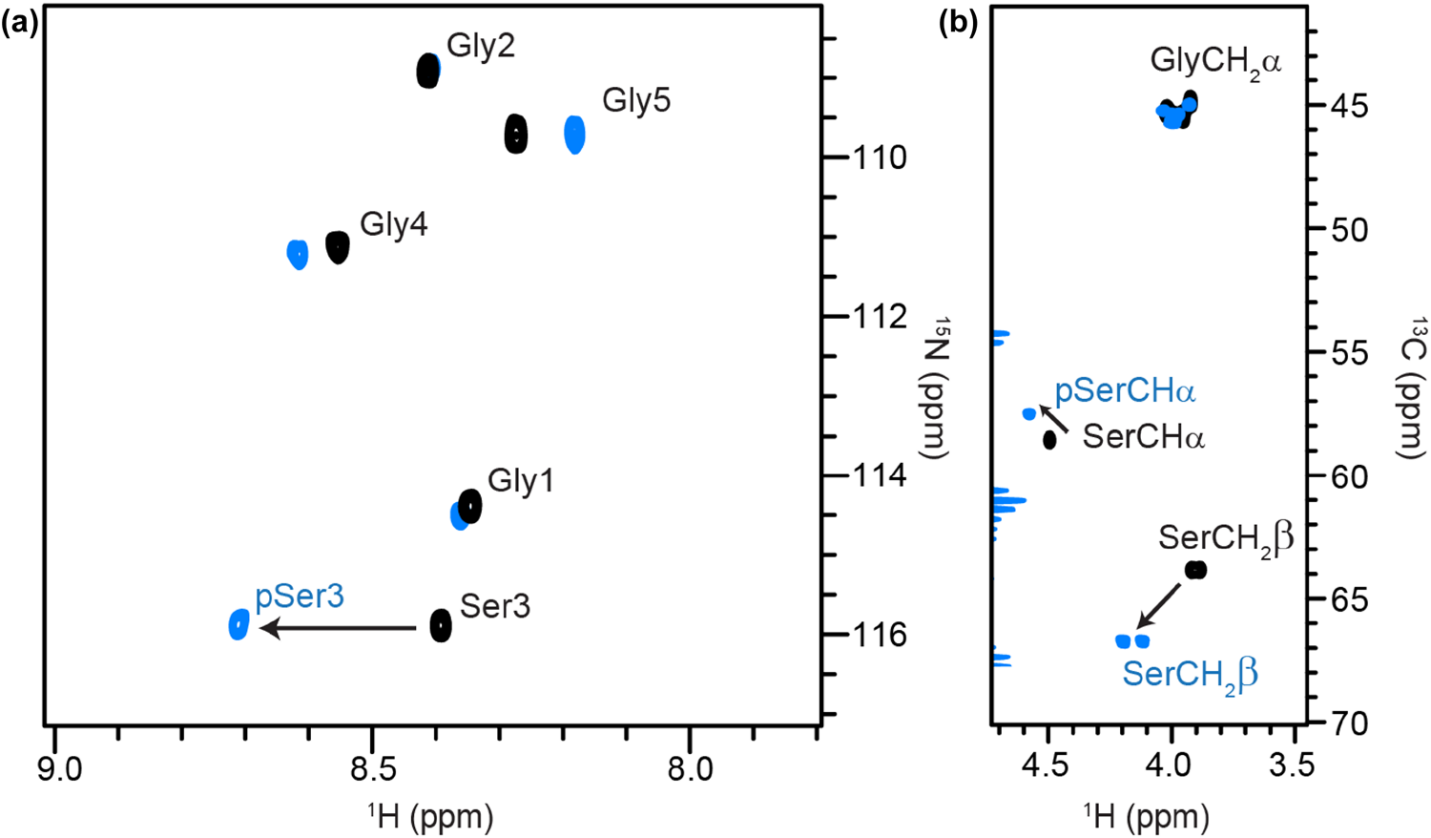
Serine phosphorylation. **a** Superposition of ^1^H–^15^N HSQC NMR spectra of random coil peptides containing serine (black contours) and phosphoserine (blue contours) as residue ‘X’. The arrow shows the characteristic ~ 0.3 ppm downfield shift of HN upon phosphorylation. **b**^1^H–^13^C HSQC NMR spectra with shifts of Hα/Cα and Hβ/Cβ marked with arrows

Phosphorylated serine, threonine and tyrosine have random coil chemical shifts (Table 1) in agreement with those reported by Bienkiewicz and Lumb (Bienkiewicz and Lumb 1999). These authors also determined the pK_a_ values of phosphoserine (pK_a_ = 5.96 ± 0.09), phosphothreonine (pK_a_ = 6.30 ± 0.07) and phosphotyrosine (pK_a_ = 5.96 ± 0.04) for the equilibrium between the singly and doubly charged phosphate groups, based on changes in the ^31^P phosphate chemical shift (Bienkiewicz and Lumb 1999; Platzer et al. 2014). Equations for calculation of chemical shifts of residues with ionisable side chains at different pH values are given in the following references (Bienkiewicz and Lumb 1999; Platzer et al. 2014; McIntosh et al. 2011). Bienkiewicz and Lumb further noted that the chemical shift changes upon phosphorylation, such as the ~ 0.3 ppm downfield shift of HN (Fig. 3), are similar to those observed when hydrogen bonds form in secondary structures (Bienkiewicz and Lumb 1999). Selenko and co-workers have demonstrated that 2D ^1^H–^15^N correlation experiments are valuable for monitoring phosphorylation events by NMR, based on this characteristic shift, which is strongly pH dependent and is due to intra-residue hydrogen bonding between the phosphate and backbone amide groups in unstructured regions (Theillet et al. 2012a; Bienkiewicz and Lumb 1999; Du et al. 2005). In a structured region or hairpin, however, the phosphate group might interact with the backbone HN of other residues nearby in space, and a shift might be observed in a non-modified residue [for examples see (Smet-Nocca et al. 2013; Bah et al. 2015; Dong et al. 2017)]. Hydrogen bonding of the phosphate moiety with either its own or other backbone amides might therefore have a significant role in the local structure and would not be present in the simple serine → glutamic acid substitution that is often used as a mimic of phosphoserine in proteins expressed recombinantly. Further evidence for the pH dependence and hydrogen bonding between the phosphate and backbone amide groups is shown by the smaller changes in chemical shifts between serine and phosphoserine when the spectra are acquired in 8 M urea and pH 2.3 (Table S4); instead of the 0.32 ppm downfield shift of HN observed in water at pH 5, a 0.21 ppm downfield shift of HN is observed in 8 M urea at pH 2.3. Downfield shifts of Hβ/Cβ resonances (~ + 0.3/~ + 3–4 ppm) occur upon phosphorylation of serine (Fig. 3) and threonine, due to the closer proximity of these nuclei to the phosphate group. Phosphorylation at serine/threonine residues preceding proline can also affect *cis/trans* isomerisation of the prolyl peptide bond (Theillet et al. 2012a).

In contrast, tyrosine phosphorylation does not cause a characteristic downfield shift of HN as observed for serine and threonine. It has been reported that the modification is most noticeable in the ^1^H–^13^C spectra by the downfield shifts of Hε/Cε (~ + 0.3/~ + 3 ppm) (Theillet et al. 2012a; Bienkiewicz and Lumb 1999). As shown in Table 1, we observed shifts of Cγ (+ 3.7 ppm), Hε/Cε (+ 0.28/+ 5.0 ppm) and Cζ (− 3.3 ppm) resonances. Although changes in the backbone chemical shifts of residues flanking phosphotyrosine have been observed in other studies and used for mapping tyrosine phosphorylation sites (Theillet et al. 2012a), we observed only a 0.17 ppm downfield shift for Gly5 HN, however this might be due to the achirality of glycine or the short peptides of only five residues used in our study.

### Sulfation of tyrosine

To our knowledge, there are no reports of NMR studies on tyrosine sulfation, in which the anionic sulfate moiety is transferred from phosphoadenosine phosphosulfate to tyrosine side chains (Walsh et al. 2005). As with tyrosine phosphorylation, this modification on the distal end of the side chain does not cause significant backbone chemical shift changes but causes changes in the aromatic nuclei shifts that are in the same direction but larger than those caused by phosphorylation: Cγ (+ 6.2 ppm), Hε/Cε (+ 0.40/+ 6.1 ppm) and Cζ (− 4.4 ppm).

### Acetylation, methylation and carboxymethylation of lysine

**Fig. 4:**
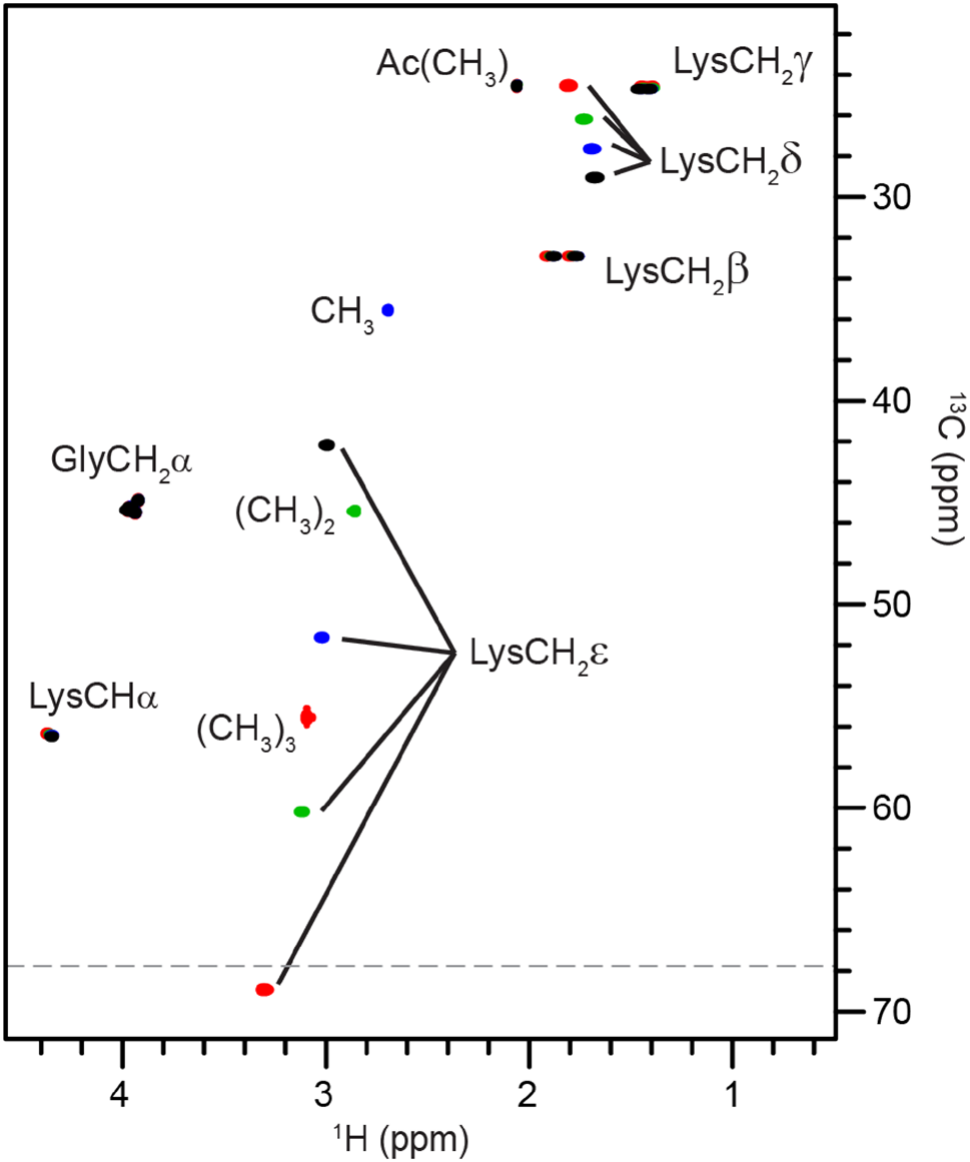
Lysine methylation. Superposition of ^1^H–^13^C HSQC NMR spectra of random coil peptides containing lysine (black contours), methyllysine (blue contours), dimethyllysine (green contours) and trimethyllysine (red contours) as residue ‘X’. The characteristic Hδ/Cδ, Hε/Cε and methyl group crosspeaks are labelled. The grey dashed line at 68 ppm indicates the wider sweep width of the ^1^H–^13^C HSQC spectrum acquired for Lys(CH_3_)_3_

Lysine acetylation converts the terminal positively charged ζNH_3_^+^ group into a neutral moiety whereas lysine methylation maintains the positive charge. In contrast, lysine carboxymethylation introduces a negatively-charged carboxylic acid, which forms a side-chain zwitterion at neutral pH (and pH 5.0 as in this study) because the alkylated ζNH_2_^+^ remains protonated. These electrostatic changes can alter interactions of the lysine side chain; for example, the interactions of histone lysine residues with negatively-charged DNA are modulated by lysine acetylation and methylation in the ‘histone code’ (Muller and Muir 2015). As has previously been noted for proteins (Theillet et al. 2012a; Liokatis et al. 2010), acetylation of lysine in the random coil peptide results in small changes in backbone HN/NH resonances (− 0.03/+ 0.5 ppm). However, the appearance of a new amide resonance for Hζ/Nζ at 7.94/127.3 ppm serves as an indicator of acetylation (Theillet et al. 2012a; Liokatis et al. 2010; Smet-Nocca et al. 2010). Small shifts in the ^1^H–^15^N crosspeaks of neighbouring residues have been used to locate lysine acetylation sites in proteins (Theillet et al. 2012a; Liokatis et al. 2010), however we only observed a change in Gly5 HN/NH of − 0.03/0.1 ppm. Whereas Hε/Cε resonances of acetyllysine appear at 3.16/42.0 ppm, those of lysine, mono-, di- and tri-methyllysine appear at 3.00/42.2 ppm, 3.03/51.6 ppm, 3.12/60.2 ppm, and 3.31/68.9 ppm, respectively (Fig. 4), allowing these different PTMs to be distinguished, as demonstrated by Thiellet et al. (2012a, b). Characteristic shifts are also observed for Hδ/Cδ and for the added methyl groups CH_3_ 2.70/35.6 ppm, (CH_3_)_2_ 2.86/45.4 ppm and (CH_3_)_3_ 3.10/55.5 ppm (Fig. 4). In contrast to acetylation and methylation, lysine carboxymethylation is a non-enzymatic PTM and is a common advanced glycation end product. The Hε/Cε resonance shifts to 3.06/50.0 ppm and an indicative ^1^H–^13^C crosspeak corresponding to the carboxymethyl CH_2_ appears at 3.60/52.0 ppm, allowing this modification to be distinguished from other lysine PTMs. The pH dependence of the ^1^H chemical shifts from pH 2–9 and the limits of the ^1^H, ^15^N and ^13^C chemical shifts were determined and are shown in Figure S1 and Table S6, respectively. Only the CH_2_ and carbonyl resonances of the carboxymethyl group showed significant pH dependence (ΔδCH_2_ = − 0.24/+ 1.3, ΔδC O = 2.0 ppm) on increasing pH. The pKa of the carboxymethyl carboxyl group was estimated to be ≤ 2 from Figure S1.

### Methylation of arginine and modification to form argpyrimidine and citrulline

Arginine can also be modified by methylation, which is an enzymatic modification, or by various non-enzymatic modifications to form advanced glycation end products such as argpyrimidine, which are found in tissues under oxidative stress. Whereas mono- and symmetric dimethylation of arginine results in very small shifts in Hδ/Cδ and the appearance of methyl resonances at 2.82/30.2 ppm and 2.81, 2.82/30.1 ppm, respectively, asymmetric dimethylation causes a downfield shift of Hδ/Cδ to 3.26/44.1 and the methyl resonances appear at 3.00/40.3 ppm. The Hε/Nε and Hη/Nη resonances also show dispersion, however these are only observed at low pH because of fast exchange with the solvent (Theillet et al. 2012a; Liepinsh and Otting 1996). In argpyrimidine, new ^13^C signals are observed for the aromatic nuclei, Hδ shifts downfield by + 0.17 ppm and the two methyl groups can be distinguished from those of dimethylarginine by their upfield shifts at 2.36/20.1 ppm. Uncharged citrulline is formed by the deimination of positively-charged arginine by peptidylarginine deiminases and this PTM can alter protein structure, binding and immunogenicity (Fuhrmann et al. 2015; Gyorgy et al. 2006). Although the mass difference of 1 Da resulting from this PTM might be difficult to detect without high resolution mass spectrometry, citrulline can easily be distinguished from arginine in ^1^H–^13^C NMR spectra by the Hγ/Cγ resonances at 1.51, 1.56/28.2 ppm and Hδ/Cδ resonances at 3.11/42.0 ppm, as well as the new carbonyl ^13^C resonance at 164.3 ppm.

### N- and O-glycosylation

The most common glycosylation PTMs in eukaryotes are O-glycosylation of serine or threonine and N-glycosylation of asparagine, and involve either simple or highly complex branched carbohydrate chains (Walsh et al. 2005). The carbohydrate moieties are typically mobile and solvent-exposed, and can modify protein structures, solubility and immunogenicity (Bourre et al. 2018; Theillet et al. 2012a). The random coil shifts of Asn(β-D-GlcNAc), with which N-glycan structures are linked to proteins, show a large downfield shift in the Hδ/Nδ resonances to 8.67/131.8 ppm in comparison to those of asparagine Hδ/Nδ (6.91, 7.61/112.8 ppm). Furthermore, the crosspeak of the H1/C1 anomeric carbon appears at 5.04/81.1 ppm, characteristic of the linkage to nitrogen in N-glycans. This anomeric ‘fingerprint’ region of N-glycosylated proteins has been used to identify glycans and analyse their composition and linkage types on a several proteins under denaturing conditions (Schubert et al. 2015). In contrast, the anomeric H1/C1 crosspeak of Thr(α-d-GalNAc) appears at 4.93/101.4 ppm, which is characteristic of an O-linked sugar. The Hβ/Cβ crosspeak of Thr(α-d-GalNAc) shifts to 4.42/78.1 ppm, while Hα/Cα shifts to 4.63/60.5 ppm, allowing threonine glycosylation to be distinguished from threonine phosphorylation. Although we did not have access to Ser(α-d-GalNAc) for comparison in this study, different orientations of the carbohydrate moiety at the glycosidic linkage have been reported for Thr(d-GalNAc) and Ser(d-GalNAc) in simple model peptides, independent of the anomeric configuration (α or β), however chemical shift assignments were not reported (Corzana et al. 2007). For O-glycosylation, the peptide backbone might be constrained in an extended conformation by interactions between the sugar and backbone (Rani and Mallajosyula 2017; Elbaum and Zondlo 2014), consistent with the changes in chemical shifts observed for Thr(GalNAc) CO (− 0.8 ppm), NH (− 1.3 ppm), Hα (+ 0.24 ppm) and Gly4 NH (− 1.7 ppm). Furthermore, in threonine ^3^J_HNHA_ = 7.5 Hz whereas in Thr(GalNAc) ^3^J_HNHA_ = 9.2 Hz, indicating a more extended conformation in the glycosylated peptide. The possible ‘extended’ rather than ‘random coil’ nature of the Thr(GalNAc) peptide should therefore be noted when using the chemical shifts as reference values, to prevent potential helical content bias.

### Hydroxylation of proline

Hydroxyproline has a key role in essential mammalian proteins such as collagen and hypoxia inducible factor 1-alpha (HIF-1α) (Walsh et al. 2005). Alongside C-terminal amidation and disulfide bond formation, it is also among the most common PTMs of conotoxins—venom peptides from cone snails that are characterised by stability, a high prevalence of PTMs and potent activity at their target receptors (Akondi et al. 2014). Hydroxylation of proline by prolyl 4-hydroxylase is the most prevalent PTM in humans and forms (2*S*,4*R*)-4-hydroxyproline (4-Hyp), which favours the *trans*-configuration and has a crucial role in stabilising the triple helix structure of collagen (Gorres and Raines 2010; Eberhardt et al. 1996). Due to the lack of an amide proton in proline and 4-hydroxyproline, the hydroxylation is most obvious in the downfield shifts of the ^1^H–^13^C crosspeaks Hβ/Cβ (+ 0.10/+ 7.7 ppm), Hγ/Cγ (+ 2.59/+ 45.4 ppm) and Hδ/Cδ (+ 0.08/+ 7.5 ppm) due to the presence of the deshielding hydroxy group on Cγ (Fig. 2). The major conformations of both the proline- and 4-hydroxyproline-containing peptides were *trans*-, with minor *cis*- conformations that could be assigned (Table 1). In agreement with the literature (Eberhardt et al. 1996), hydroxylation at the 4*R* position increases the population of the *trans*- configuration (Pro ~ 11.5% *cis*-, 4-Hyp ~ 9% *cis*-). These *cis*- populations are likely to be higher in the random coil peptides than in natural proteins, however, due to the minimal constraints of the neighbouring glycine residues. Also in agreement with literature (Marx et al. 2006), in the *cis*- conformation of 4-hydroxyproline, there is a smaller chemical shift difference between the Cβ and Cγ resonances (28.3 ppm) than in the *trans*-configuration (32.9 ppm).

### γ-Carboxylation of glutamic acid

**Fig. 5:**
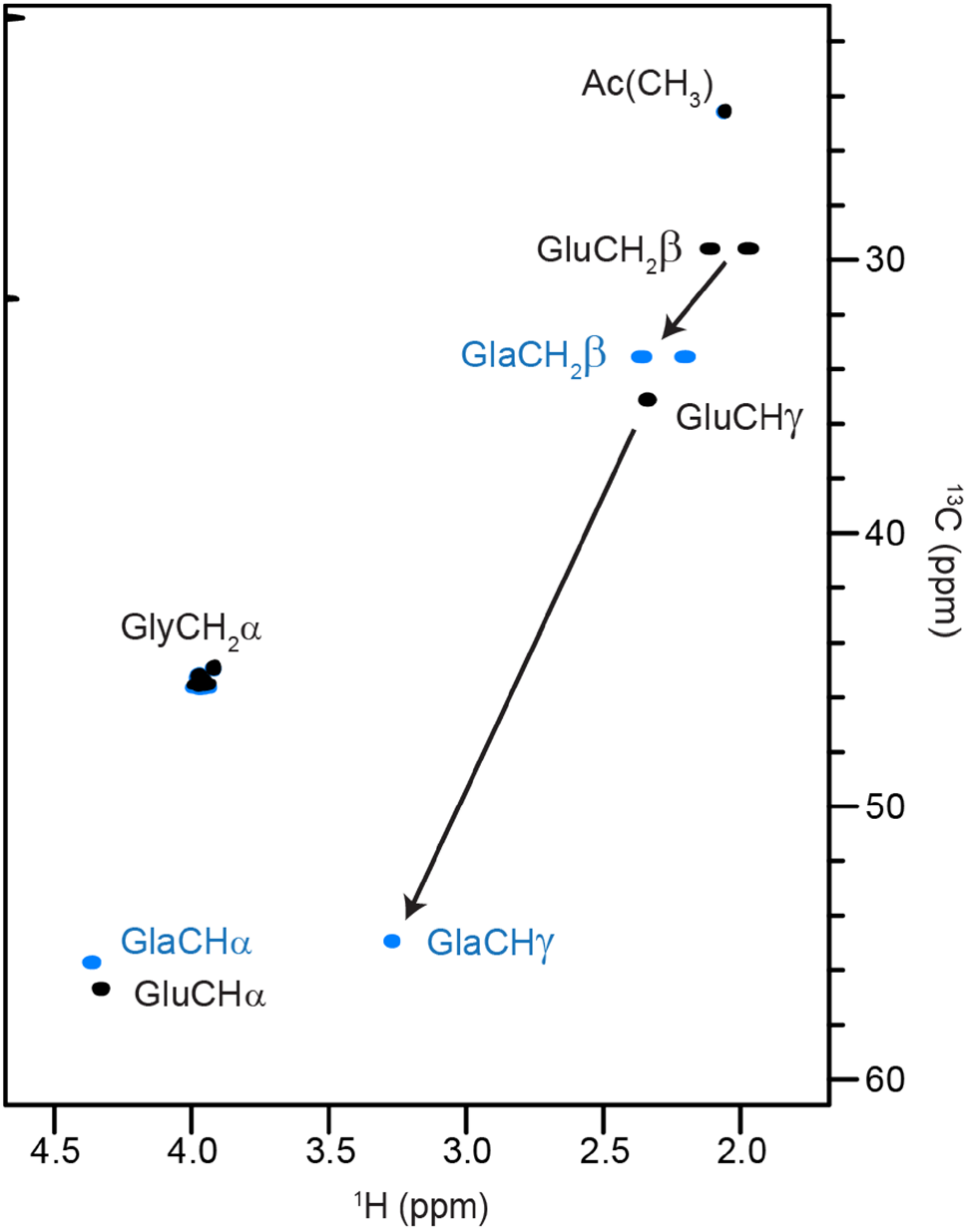
Glutamic acid γ-carboxylation. Superposition of ^1^H–^13^C HSQC NMR spectra of random coil peptides containing glutamic acid (black contours) and γ-carboxyglutamate (blue contours) as residue ‘X’. The arrows show the characteristic downfield shifts of Hβ/Cβ and Hγ/Cγ upon carboxylation

Also common in conotoxins and having an important role in the folding of blood clotting factor coagulation protease factor IX (Walsh et al. 2005), γ-carboxylation of glutamic acid causes downfield shifts of the Hβ/Cβ resonances (+ 0.23/+ 3.9 ppm) and Hγ/Cγ resonances (+ 0.93/+ 19.8 ppm), making this modification easy to identify (Figs. 2 and 5). This PTM is often associated with Ca^2+^ binding and induction of helicity and several NMR structures of conotoxins containing γ-carboxyglutamate have been published with Ca^2+^ bound, however, the chemical shifts vary only marginally compared to those without Ca^2+^ bound (Marx et al. 2006). In contrast to the random coil Hα shift of γ-carboxyglutamate (4.37 ppm, Table 1), the average Hα shift of γ-carboxyglutamate in the conotoxin structures reviewed by Marx et al. is 4.16 ppm, which is due to its propensity to be found in helices (Marx et al. 2006). This illustrates the difference between the random coil shifts reported here and average shifts from structural databases and, moreover, how deviations from random coil shifts can indicate the presence of secondary structures. Similarly to serine and threonine phosphorylation discussed above, the additional carboxyl group in γ-carboxyglutamate might also interact with nearby residues in space and cause changes in their chemical shifts. The pH dependence of the ^1^H chemical shifts from pH 2–9 and the limits of the ^1^H, ^15^N and ^13^C chemical shifts were determined and are shown in Figure S2 and Table S6, respectively. Chemical shift dependence on pH is observed for all the ^1^H resonances but is most noticeable in the Hγ/Cγ shifts (ΔδHγCγ = 0.39/6.0 ppm) and the two side chain carbonyls (ΔδCO = 5.2 ppm). The titration curve appears to be biphasic, with pKa values of ~ 2.7 and 4.6. More detailed measurements would be needed to study the linked equilibria of the two carboxyl groups, as has been demonstrated for two glutamic acid residues in *Bacillus circulans* xylanase (McIntosh et al. 2011).

### Flanking glycine residues

The chemical shifts of the glycine residues flanking the residue ‘X’ of interest are given in Table S3. The chemical shifts of the flanking residues are not reported in other random coil datasets (with the exception of a study of neighbour effects by Schwarzinger et al. 2001), but we have included them as they provide information about the internal consistency of the dataset and the effects of modifications on neighbouring residues. As shown in Table S3, the standard deviations, particularly of the resonances belonging to the terminal residues show very high consistency of the chemical shifts (0.00 ppm ≤ std. dev. ^1^H ≤ 0.09 ppm; 0.0 ppm ≤ std. dev. ^13^C ≤ 0.2 ppm; 0.1 ppm ≤ std. dev. ^15^N ≤ 0.7 ppm), despite slight variations in pH and concentration between the samples (Table S2). As would be expected from the proximity of the variable residue ‘X’ (residue 3), the highest standard deviations are observed for the resonances of residues Gly2 and Gly4. The Gly2 HN, Cα and CO resonances are all upfield shifted and the Hα resonances downfield shifted when followed by proline or hydroxyproline, in agreement with observations made by Wishart et al. (1995) Gly4 HN resonances are downfield shifted when preceded by phosphoserine or phosphothreonine (+ 0.07 ppm), and hydroxyproline (+ 0.13 ppm), whereas the Gly4 NH is upfield shifted (− 1.7 ppm) when preceded by Thr(GalNAc). When residue ‘X’ is tyrosine, phosphotyrosine or sulfotyrosine, Gly5 HN resonances are upfield shifted, particularly for tyrosine (− 0.34 ppm, compared to the average shift of Gly5 HN). As shown by Wishart et al., the neighbouring residues can affect chemical shifts of the residue of interest as they influence the φ and ψ angle preferences (Wishart et al. 1995). For example, ^15^NH shifts can be affected by the preceding residue (*i *− 1) by up to 4.5 ppm because ^15^N shifts are correlated with the ψ angle of the preceding residue (Wishart and Nip 1998). In contrast, the following residue (*i *+ 1), particularly proline and aromatic amino acids, tends to affect Hα and Cα chemical shifts (Wishart and Nip 1998). Although glutamine has been suggested as a flanking residue that might provide better predictions of Ramachandran distribution (Ting et al. 2010; Kjaergaard and Poulsen 2011), we have used glycine as both preceding and following residues to allow for maximum conformational flexibility and for compatibility with other random coil datasets, allowing for broad application. Our observed random coil shifts of the standard amino acids are nevertheless in good agreement with the random coil shifts of residues followed by alanine (Wishart et al. 1995).

## Conclusion

We have synthesised a set of random coil peptides containing common naturally-occurring protein PTMs and assigned the random coil NMR chemical shifts. We have highlighted distinctive NMR signatures of the PTMs and compared them with values reported in the literature. The random coil shifts can be used in conjunction with established random coil shifts of the standard amino acids to identify PTMs in proteins and peptides and to determine the presence of structural elements or structural propensity. Furthermore, the random coil shifts of the modified residues determined here can be added to the reference datasets of programs that use secondary chemical shifts to predict structural parameters or structures of proteins containing PTMs. This will help us to understand the role of PTMs in protein structure and function and will provide insights into protein regulation by PTMs in both normal cellular processes and disease.

## Electronic supplementary material

Below is the link to the electronic supplementary material.

Supplementary material 1—Synthesis details, NMR sample conditions, chemical shifts of the flanking residues and comparison with literature values are available in the attached supporting information (PDF 942 kb)
