# Supplementary material for: Random coil shifts of posttranslationally modified amino acids
Source: J Biomol NMR. 2019 Jul 17;73(10):587–99. doi: 10.1007/s10858-019-00270-4 (PMC6859290; doi:10.1007/s10858-019-00270-4)
Supplement: Supplementary file 1 — Supplementary material 1—Synthesis details, NMR sample conditions, chemical shifts of the flanking residues and comparison with literature values are available in the attached supporting information (PDF 942 kb) [file 10858_2019_270_MOESM1_ESM.pdf]

Supplementary Information

Random coil shifts of posttranslationally modified amino acids

Anne C. Conibear[1,2*], K. Johan Rosengren[2], Christian F. W. Becker[1], and Hanspeter Kaehlig[3]

[1] University of Vienna, Faculty of Chemistry, Institute of Biological Chemistry, Währinger Straße 38, 1090 Vienna, Austria.

[2] The University of Queensland, School of Biomedical Sciences, QLD 4072, Brisbane, Australia.

[3] University of Vienna, Faculty of Chemistry, Institute of Organic Chemistry, Währinger Straße 38, 1090 Vienna, Austria.

ORCIDs

Anne C. Conibear: 0000-0002-5482-6225

Christian F. W. Becker: 0000-0002-8890-7082

Hanspeter Kaehlig: 0000-0002-3898-6501

K. Johan Rosengren: 0000-0002-5007-8434

[*]Corresponding author:

Anne C. Conibear

The University of Queensland, School of Biomedical Sciences, QLD 4072, Brisbane, Australia.

E-mail: a.conibear@uq.edu.au

Phone: + 61-7-3365-1738

Table S1. Building blocks, suppliers and synthesis procedures for Fmoc-SPPS of random coil peptides containing modified amino acids.

Residue 'X' and building block	Supplier (Catalogue No.)	Notes[a, b]
phosphoserine Fmoc-Ser(PO(OBzl)OH)-OH	CEM (#A040-A)	Both manual SPPS and adjusted procedure for microwave synthesis[c] used. Standard cleavage conditions.
phosphothreonine Fmoc-Thr(PO(OBzl)OH)-OH	CEM (#A041-A)	Both manual SPPS and adjusted procedure for microwave synthesis[c] used. Standard cleavage conditions.
phosphotyrosine Fmoc-Tyr(PO(OBzl)OH)-OH or Fmoc-Tyr(PO(NMe_2)$_2$-OH	CEM (#A042-A) Merck (852090)	Synthesized on Liberty Blue microwave synthesizer (CEM).[b] Standard cleavage conditions. Dimethylamine removed by acid catalyzed hydrolysis - incubated in 10% TFA for 48 h, then lyophilized.
acetyllysine Fmoc-Lys(ac)-OH	Merck (852042)	Standard coupling and cleavage conditions.
dimethylarginine (symmetric) Fmoc-SDMA.HCl or Fmoc-SDMA(Boc)$_2$-ONa	Bachem (B-3345.0001) Merck (852310)	Fmoc-SDMA.HCl used. Synthesised on Liberty Blue microwave synthesizer (CEM). Standard coupling and cleavage conditions.
dimethylarginine (asymmetric) Fmoc-ADMA(Pbf)-OH	VWR	Standard coupling and cleavage conditions.
methylarginine Fmoc-Arg(Me,Pbf)-OH	GL Biochem (36417)	Standard coupling and cleavage conditions.
trimethyllysine Fmoc-Lys(Me)$_3$-OH	GL Biochem (21152)	Standard coupling and cleavage conditions.
dimethyllysine Fmoc-Lys(Me)$_2$-OH.HCl	GL Biochem (22703)	Standard coupling and cleavage conditions.
methyllysine Fmoc-Lys(Me,Boc)-OH	GL Biochem (36816)	Standard coupling and cleavage conditions.
argpyrimidine Fmoc-Apy(Pbf,TBMS)-OH	Internal[d]	TBMS was replaced by acetylation during N-terminal acetylation of the peptide. Acetyl group on Apy was removed on resin with NaOMe (10 mM) in DMF/MeOH 17:3 for 2 h, followed by standard cleavage.
4(*R*)-hydroxyproline Fmoc-Hyp(tBu)-OH	Merck (852036)	Synthesised on Liberty Blue microwave synthesizer (CEM). Standard coupling and cleavage conditions.
citrulline Fmoc-Cit-OH	GL Biochem (21602)	Standard coupling and cleavage conditions.

threonine(GalNAc) Fmoc-Thr(α-D-GalNAc(Ac)$_3$)-OH	Sussex research (GA131000)	Thr(GalNAc) (1.2 eq.) coupled with HATU (1.2 eq.), DIPEA (2.5 eq.) for 2 h. GalNAc deprotected on resin with 10 mM NaOMe in DMF/MeOH 17:3 for 1 h. Cleavage for 1 h with standard cleavage cocktail to minimize cleavage of GalNAc.
asparagine(GlcNAc) Fmoc-Asn(β-D-GlcNAc(Ac)$_3$)-OH	Sigma (8521358500)	Asn(GlcNAc) (2 eq.) coupled with Oxyma (2 eq.) and DIC (2.2 eq.) for 2 h. Sugar deprotected on resin with 10 mM NaOMe in DMF/MeOH 17:3 for 1 h. Cleavage for 1 h with standard cleavage cocktail.
sulfotyrosine Fmoc-Tyr(SO$_3$.nP)-OH	Merck (852347)	Synthesised on Sieber amide resin with standard couplings. Cleaved with 1% TFA in DCM (3 x 5 min). DCM evaporated. Neopentyl group removed with 2 M ammonium acetate, pH 7.2 at 37 °C for 5 h and then lyophilized several times. ESI-MS in negative ion mode.
gammacarboxyglutamate Fmoc-Gla(OtBu)$_2$-OH	Iris Biotech (FAA1368)	Standard coupling and cleavage conditions.
carboxymethyllysine Fmoc-CML(OtBu)(Boc)-OH	Iris Biotech (FAA3620)	Standard coupling and cleavage conditions.

a. Unless otherwise noted, peptides were synthesized manually using the standard coupling and cleavage conditions described in the Materials and Methods section.

b. Standard coupling procedure for automated microwave peptide synthesis (Liberty Blue, CEM): Syntheses were carried out at 0.05 mmol scale on Rink amide resin. Couplings were carried out with diisopropyl carbodiimide (DIC, 0.25 M in DMF) and Oxyma (0.5 M in DMF) to couple the amino acids (5 eq.) Couplings were carried out for 4 min at 90 °C. N-terminal acetylation was achieved with 10% v/v acetic anhydride in DMF 2 x 5 min. Cleavage and analysis was carried out as for manually synthesized peptides as described in the Materials and Methods.

c. Adjusted procedure for microwave synthesis of peptides containing phosphorylated residues (CEM application notes): After incorporation of a phospho residue, the next Fmoc removal was carried out at room temperature (most important for phosphoserine). DIPEA (0.4 eq.) was added to the coupling mixture for phosphopeptides by adding it to the Oxyma in DMF bottle. Coupling times were 4 min at 90 °C.

d. Matveenko, M., Cichero, E., Fossa, P. & Becker, C.F. Impaired Chaperone Activity of Human Heat Shock Protein Hsp27 Site-Specifically Modified with Argpyrimidine. *Angew. Chem. Int. Ed. Engl.* **55**, 11397-402 (2016).

Table S2. Samples of random coil peptides used for NMR spectroscopy.

Residue 'X'	NMR sample (mg)[a]	NMR sample pH
phosphoserine	4.3	5.2
phosphothreonine	5.3	4.9
phosphotyrosine	5.5	5.2
acetyllysine	4.0	5.2
dimethylarginine (symmetric)	4.5	4.7
dimethylarginine (asymmetric)	4.7	5.0
methyl arginine	4.7	4.8
trimethyllysine	4.6	4.8
dimethyllysine	4.8	4.8
monomethyllysine	5.0	5.0
argpyrimidine	5.2	4.8
4-hydroxyproline	5.3	5.0
citrulline	5.0	4.8
threonine(GalNAc)	4.9	4.8
asparagine(GlcNAc)	4.9	4.9
sulfo-tyrosine	7.3	5.2
gammacarboxyglutamate	4.8	4.8
carboxymethyllysine	4.9	5.1
serine	3.0	5.2
threonine	5.0	4.7
tyrosine	4.5	5.3
lysine	5.0	5.3
arginine	4.7	5.3
proline	4.6	4.9
asparagine	4.7	5.0
glutamic acid	4.1	4.7
glycine	4.9	5.3

a. Sample volume 600 μL H_2O/D_2O 9:1

Table S3. Chemical shifts of glycine residues flanking residue 'X'.

	Ac			Gly1					Gly2					X	Gly4					Gly5					NH2	
X'	H_3C	CH_3	CO	NH	HN	Hα	Cα	CO	NH	HN	Hα	Cα	CO		NH	HN	Hα	Cα	CO	NH	HN	Hα	Cα	CO	H_2N	NH_2
Ser	2.06	24.5	177.9	114.4	8.35	3.96	45.5	175.3	108.9	8.41	4.03	45.3	174.6		111.1	8.56	4.00	45.5	174.8	109.7	8.28	3.93	44.9	177.0	7.09<br>7.52	107.3
pSer	2.06	24.6	177.8	114.5	8.37	3.98	45.4	175.3	108.9	8.41	4.04	45.2	174.8		111.2	8.63	4.00	45.7	174.9	109.7	8.19	3.93	45.0	177.1	7.08<br>7.50	107.3
Thr	2.06	24.5	177.8	114.4	8.35	3.96	45.5	174.9	108.9	8.44	4.05	45.3	174.9		111.5	8.56	4.00	45.5	174.8	109.8	8.30	3.93	44.9	177.0	7.09<br>7.53	107.3
pThr	2.06	24.5	177.7	114.4	8.36	3.98	45.3	175.2	108.9	8.41	4.05	45.0	174.9		111.3	8.63	4.00	45.8	175.0	110.1	8.32	3.92	45.0	177.1	7.08<br>7.52	107.3
Thr(GalNAc)	2.07	24.6	177.8	114.0	8.33	3.95	45.5	175.2	108.6	8.42	4.09	45.3	174.9		109.8	8.50	3.97<br>4.00	45.4	174.5	109.4	8.37	3.92	44.8	177.0	7.07<br>7.55	107.3
Tyr	2.06	24.5	177.8	114.2	8.31	3.91	45.4	175.1	108.5	8.31	3.91	45.4	174.2		111.5	8.48	3.90	45.0	174.6	109.3	7.92	3.89	45.0	176.9	7.09<br>7.49	107.2
pTyr	2.06	24.6	177.8	114.3	8.33	3.91	45.4	175.1	108.5	8.32	3.88	45.1	174.3		111.5	8.50	3.88<br>3.94	45.5	174.7	109.7	8.09	3.91	44.9	177.1	7.10<br>7.50	107.3
Tyr(SO_3)	2.06	24.5	177.9	114.2	8.28	3.91	45.3	175.1	108.4	8.29	3.89	45.0	174.3		111.5	8.49	3.88<br>3.94	45.5	174.7	109.6	8.10	3.91	44.9	176.9	7.08<br>7.49	107.2
Lys	2.06	24.6	177.9	114.3	8.36	3.94	45.5	174.7	108.7	8.42	3.97	45.3	174.7		110.3	8.54	3.98	45.3	174.6	109.8	8.29	3.93	44.9	177.0	7.09<br>7.53	107.2
Lys(ac)	2.06	24.5	177.8	114.3	8.35	3.94	45.5	175.2	108.7	8.40	3.97	45.2	174.7		110.3	8.55	3.97	45.2	174.6	109.7	8.26	3.92	44.9	177.0	7.09<br>7.51	107.2
Lys(Me)	2.06	24.5	177.9	114.3	8.36	3.94	45.5	175.3	108.7	8.42	3.97	45.3	174.6		110.3	8.54	3.98	45.3	174.7	109.8	8.30	3.93	44.9	177.0	7.09<br>7.53	107.2
Lys$(Me)_2$	2.06	24.5	177.9	114.4	8.36	3.95	45.5	174.6	108.7	8.42	3.97	45.3	174.6		110.3	8.54	3.98	45.3	174.8	109.8	8.30	3.93	44.9	177.0	7.09<br>7.53	107.2
Lys$(Me)_3$	2.06	24.6	177.9	114.3	8.35	3.94	45.5	175.3	108.6	8.41	3.97	45.3	174.6		110.3	8.54	3.98	45.4	174.7	109.8	8.30	3.93	44.9	176.9	7.09<br>7.53	107.2
CML	2.06	24.5	177.9	114.3	8.35	3.95	45.6	175.3	108.7	8.40	3.98	45.3	174.6		110.3	8.54	3.98	45.3	174.8	109.7	8.27	3.93	45.0	177.1	7.08<br>7.52	107.2
Arg	2.06	24.5	177.9	114.4	8.37	3.95	45.5	175.3	108.7	8.43	3.98	45.4	174.6		110.3	8.55	3.98	45.4	174.7	109.8	8.30	3.93	44.9	177.0	7.08<br>7.53	107.3
Arg(Me)	2.06	24.5	178.0	114.3	8.36	3.94	45.5	175.3	108.7	8.43	3.98	45.3	174.6		110.3	8.54	3.98	45.3	174.5	109.7	8.30	3.93	44.9	177.0	7.08<br>7.52	107.2
SDMA	2.06	24.5	177.9	114.3	8.36	3.94	45.5	175.3	108.8	8.43	3.98	45.4	174.6		110.4	8.54	3.98	45.3	174.6	109.7	8.30	3.93	44.9	177.1	7.09<br>7.53	107.3
ADMA	2.06	24.5	177.9	114.3	8.36	3.94	45.5	175.3	108.7	8.42	3.98	45.3	174.6		110.3	8.54	3.98	45.3	174.6	109.7	8.29	3.93	44.9	176.9	7.09<br>7.53	107.2
Apy	2.05	24.5	177.8	114.3	8.34	3.93	45.5	175.2	108.7	8.40	3.97	45.3	174.6		110.2	8.54	3.96	45.5	174.7	109.7	8.26	3.92	44.9	177.0	7.08<br>7.51	107.2

Pro	2.06	24.5	177.7	114.3	8.31	3.96	45.3	174.9	108.8	8.25	4.13	44.5	172.3	109.8	8.63	3.97	45.4	174.8	109.7	8.23	3.92	44.9	177.0	7.09<br>7.49	107.2
4-Hyp	2.06	24.6	177.7	114.2	8.31	3.96	45.2	174.8	108.5	8.26	4.09<br>4.15	44.6	172.6	110.5	8.76	3.98	45.5	174.8	109.7	8.24	3.93	44.9	177.0	7.09<br>7.48	107.2
Asn	2.06	24.5	177.8	114.3	8.33	3.95	45.4	175.2	108.7	8.41	3.98	45.3	174.7	109.8	8.53	3.98	45.6	174.8	109.7	8.27	3.93	44.9	177.0	7.09<br>7.50	107.3
Asn(GlcNAc)	2.06	24.5	177.8	114.2	8.33	3.95	45.4	175.3	108.7	8.39	3.98	45.4	174.4	109.3	8.48	3.94<br>3.99	45.6	174.8	109.6	8.26	3.93	44.9	177.0	7.09<br>7.51	107.3
Glu	2.06	24.6	177.8	114.5	8.36	3.96	45.5	175.3	108.8	8.41	3.98	45.2	174.6	110.3	8.59	3.98	45.5	174.8	109.8	8.27	3.93	44.9	177.0	7.09<br>7.51	107.3
Gla	2.06	24.6	177.8	114.5	8.37	3.98	45.5	175.3	108.8	8.41	3.98	45.2	174.6	110.2	8.59	3.98	45.6	174.9	109.8	8.29	3.93	44.9	177.1	7.09<br>7.50	107.3
Cit	2.06	24.5	177.9	114.3	8.34	3.94	45.5	175.2	108.8	8.39	3.98	45.2	174.6	110.2	8.54	3.97	45.4	174.8	109.7	8.27	3.93	44.9	177.0	7.08<br>7.51	107.2
Gly	2.06	24.5	177.9	114.4	8.36	3.97	45.4	175.4	108.9	8.45	4.01	45.5	175.0	109.0	8.40	3.99	45.3	174.9	109.8	8.35	3.93	44.9	177.0	7.08<br>7.52	107.3
Ave	2.06	24.5	177.8	114.3	8.34	3.95	45.4	175.2	108.7	8.39	3.99	45.2	174.5	110.4	8.55	3.98	45.4	174.7	109.7	8.26	3.93	44.9	177.0	7.30	107.2
Std Dev	0.00	0.0	0.1	0.1	0.02	0.02	0.1	0.2	0.1	0.04	0.05	0.1	0.2	0.7	0.05	0.02	0.2	0.1	0.1	0.09	0.01	0.0	0.1	0.02	0.1

Table S4. Random coil chemical shifts of peptides in 8M urea, pH 2.3, 10% D_2O, 298 K.

Residue 'X'	NH	HN	Hα	Hβ	Cα	Cβ	C=O	Others
Ser (water, pH 5)	115.9	8.40	4.50	3.88, 3.93	58.6	63.8	175.5	
Ser (8M urea, pH 2.3)	n.d.	8.40	4.51	3.89, 3.95	58.4	63.8	n.d.	
pSer (water, pH 5)	115.9	8.72	4.58	4.12, 4.20	57.6	66.7	175.1	
pSer (8M urea, pH 2.3)	n.d.	8.61	4.63	4.17, 4.25	57.1	66.8	n.d.	
Lys (water, pH 5)	120.9	8.31	4.35	1.78, 1.88	56.5	32.9	177.5	Hγ 1.41, 1.46, Cγ 24.7; Hδ 1.68, Cδ 29.0; Hε 3.00, Cε 42.2
Lys (8M urea, pH 2.3)	n.d.	8.33	4.35	1.78, 1.88	56.6	33.1	n.d.	Hγ 1.42, 1.47, Cγ 24.9; Hδ 1.68, Cδ 29.3; Hε 3.00, Cε 42.3
Lys(ac) (water, pH 5)	121.4	8.28	4.31	1.74, 1.84	56.8	33.1	177.7	Hγ 1.34, 1.39, Cγ 25.1; Hδ 1.51, Cδ 30.5; Hε 3.16, Cε 42.0; Hζ 7.94, Nζ 127.3; Ac(CO 176.8; H_3C 1.97, CH_3 24.6)
Lys (ac) (8M urea, pH 2.3)	n.d.	8.29	4.32	1.74, 1.84	56.9	33.3	n.d.	Hγ 1.35, 1.39, Cγ 25.3; Hδ 1.51, Cδ 30.7; Hε 3.16, Cε 42.1; Hζ 7.82; Ac(H_3C 1.98, CH_3 24.7)

Table S5. Comparison of measured random coil shifts with literature values[a]

Residue	Reference	NH	HN	Hα	Hβ	Cα	Cβ	C=O	Others
Ser	This study	115.9	8.40	4.50	3.88, 3.93	58.6	63.8	175.5	
	Wishart, 1995[b]	115.7	8.31	4.47	3.87, 3.89	58.3	63.8	174.6	
	Schwarzinger, 2000[c]	115.5	8.43	4.51	3.90, 3.95	58.7	64.1	175.4	
	Kjaergaard, 2011[d]	117.6	8.59	4.44	-	58.6	63.7	174.8	
Thr	This study	113.1	8.22	4.39	4.31	62.0	69.8	175.7	Hγ 1.21, Cγ 21.5
	Wishart, 1995[b]	113.6	8.15	4.35	4.24	61.8	69.8	174.7	Hγ 1.21, Cγ 21.5
	Schwarzinger, 2000[c]	112.0	8.25	4.43	4.33	62.0	70.0	175.6	
	Kjaergaard, 2011[d]	116.4	8.41	4.34	-	62.2	69.9	174.7	
Tyr	This study	120.5	8.21	4.57	2.98, 3.08	58.2	38.7	176.8	Cγ 130.7; Hδ 7.15, Cδ 133.3; Hε 6.85, Cε 118.3; Cζ 157.3
	Wishart, 1995[b]	120.3	8.12	4.55	2.98, 3.03	57.9	38.8	175.9	Cγ 130.6; Hδ 7.14, Cδ 133.3; Hε 6.84, Cε 118.2; Cζ 157.3
	Schwarzinger, 2000[c]	120.9	8.26	4.58	2.97, 3.09	58.3	38.9	176.7	
	Kjaergaard, 2011[d]	121.8	8.40	4.59	-	58.0	38.7	175.8	
Lys	This study	120.9	8.31	4.35	1.78, 1.88	56.5	32.9	177.5	Hγ 1.41, 1.46, Cγ 24.7; Hδ 1.68, Cδ

									29.0; Hε 3.00, Cε 42.2
	Wishart, 1995[b]	120.4	8.29	4.32	1.75, 1.84	56.2	33.1	176.6	Hγ 1.44, 1.44, Cγ 24.7; Hδ 1.68, Cδ 29.0; Hε 2.99, Cε 41.9; ζH_3N^+ 7.81; ζNH_3^+ 125.9
	Schwarzinger, 2000[c]	121.6	8.36	4.36	1.77, 1.89	56.7	33.2	177.4	
	Kjaergaard, 2011[d]	123.6	8.59	4.29	-	56.5	33.0	176.7	
Arg	This study	120.7	8.34	4.37	1.78, 1.91	56.3	30.6	177.3	Hγ 1.62, 1.66, Cγ 27.1; Hδ 3.21, Cδ 43.3; Hε 7.20; Cζ 159.6
	Wishart, 1995[b]	120.5	8.23	4.34	1.76, 1.86	56.0	30.9	176.3	Hγ 1.63, 1.63, Cγ 27.1; Hδ 3.20, Cδ 43.3; Hε 8.07; Cζ 159.5
	Schwarzinger, 2000[c]	121.2	8.39	4.38	1.79, 1.91	56.5	30.9	177.1	
	Kjaergaard, 2011[d]	123.2	8.61	4.32	-	56.3	30.8	176.4	
Pro	This study	-	-	4.45	1.99, 2.29	63.7	32.0	178.1	Hγ 2.04, Cγ 27.2; Hδ 3.64, 3.67, Cδ 49.8
	Wishart, 1995[b]	-	-	4.42	1.94, 2.29	63.3	32.1	177.3	Hγ 2.02, Cγ 27.2; Hδ 3.63, 3.63, Cδ 49.8
	Schwarzinger, 2000[c]	-	-	4.45	1.99, 2.29	63.7	32.2	177.8	

	Kjaergaard, 2011[d]	-	-	4.44	-	63.2	32.2	177.0	
Asn	This study	118.8	8.47	4.76	2.80, 2.86	53.3	38.9	176.3	Cγ 177.4; H_2Nδ 6.91, 7.61, NH_2δ 112.8
	Wishart, 1995[b]	118.7	8.40	4.74	2.75, 2.83	53.1	38.9	175.2	Cγ 177.2; H_2Nδ 6.91, 7.59, NH_2δ 112.7
	Schwarzinger, 2000[c]	119.0	8.51	4.79	2.81, 2.88	53.3	39.1	176.1	
	Kjaergaard, 2011[d]	120.0	8.67	4.70	-	53.4	38.6	175.4	
Glu	This study	120.7	8.50	4.34	1.98, 2.11	56.7	29.6	177.3	Hγ 2.34, Cγ 35.1; Cδ 182.8
	Wishart, 1995[b]	120.2	8.42	4.35	1.96, 2.06	56.6	29.9	176.6	Hγ 2.31, Cγ 35.6; Cδ 183.4
	Schwarzinger, 2000[c]	120.2	8.40	4.42	2.01, 2.18	56.1	29.9	176.8	
	Kjaergaard, 2011[d]	122.9	8.69	4.27	-	56.8	30.2	176.7	
Gly	This study	109.1	8.42	4.01	-	45.5	-	175.1	
	Wishart, 1995[b]	108.8	8.33	3.96	-	45.1	-	174.9	
	Schwarzinger, 2000[c]	107.5	8.41	4.02	-	45.4	-	174.9	
	Kjaergaard, 2011[d]	110.6	8.66	3.98	-	45.3	-	174.4	

[a] Random coil shift values are given as those reported in the respective studies and are uncorrected for differences in peptide design, pH, temperature or solution components. Conditions for each study are given below. Equations to adjust chemical shifts for temperature (1), pH (2) and neighbor effects (3) are given in:

1) Merutka, G., Dyson, H.J. & Wright, P.E. 'Random coil' 1H chemical shifts obtained as a function of temperature and trifluoroethanol concentration for the peptide series GGXGG. *J. Biomol. NMR* **5**, 14-24 (1995). Kjaergaard, M., Brander, S. & Poulsen, F.M. Random coil chemical shift for intrinsically disordered proteins: effects of temperature and pH. *J. Biomol. NMR* **49**, 139-49 (2011).
2) Platzer, G., Okon, M. & McIntosh, L.P. pH-dependent random coil (1)H, (13)C, and (15)N chemical shifts of the ionizable amino acids: a guide for protein pK a measurements. *J. Biomol. NMR* **60**, 109-29 (2014).
McIntosh, L.P., Naito, D., Baturin, S.J., Okon, M., Joshi, M.D. & Nielsen, J.E. Dissecting electrostatic interactions in Bacillus circulans xylanase through NMR-monitored pH titrations. *J. Biomol. NMR* **51**, 5-19 (2011).
3) Schwarzinger, S., Kroon, G.J., Foss, T.R., Chung, J., Wright, P.E. & Dyson, H.J. Sequence-dependent correction of random coil NMR chemical shifts. *J. Am. Chem. Soc.* **123**, 2970-8 (2001). Kjaergaard, M. & Poulsen, F.M. Sequence correction of random coil chemical shifts: correlation between neighbor correction factors and changes in the Ramachandran distribution. *J. Biomol. NMR* **50**, 157-65 (2011).

[b] Wishart, D.S., Bigam, C.G., Holm, A., Hodges, R.S. & Sykes, B.D. ^{1}H, ^{13}C and ^{15}N random coil NMR chemical shifts of the common amino acids. I. Investigations of nearest-neighbor effects. *J. Biomol. NMR* **5**, 67-81 (1995).
Random coil shifts for residue ‘X’ when followed by alanine in the peptides Ac-GGXAGG-NH_2. Conditions: 5-7 mg of peptide dissolved in 500 μL of 99.9% D_2O, 1.0 M deuterated urea, 50 mM phosphate, pH 5.0 ± 0.3, 25 °C, referenced to DSS.

[c] Schwarzinger, S., Kroon, G.J., Foss, T.R., Wright, P.E. & Dyson, H.J. Random coil chemical shifts in acidic 8 M urea: implementation of random coil shift data in NMRView. *J. Biomol. NMR* **18**, 43-8 (2000).
Peptides: Ac-GGXGG-NH_2 Conditions: 5-8 mg of peptide dissolved in 0.5 mL of 8 M urea, pH 2.3 ± 0.05 containing 10% D_2O and 10 mM DSS at 293 K.

[d] Kjaergaard, M. & Poulsen, F.M. Sequence correction of random coil chemical shifts: correlation between neighbor correction factors and changes in the Ramachandran distribution. *J. Biomol. NMR* **50**, 157-65 (2011).
Peptides: Ac-QQXQQ-NH_2 Conditions: 2-3 mg peptide dissolved in 500 μL 20 mM sodium phosphate buffer, pH 6.5 containing 5% (v/v) D_2O, 3 mM NaN_3 and 1 mm DSS at 5 °C.

Table S6. Chemical shifts at pH limits for pH titrations of γ-carboxy glutamic acid (Gla) and carboxymethyl lysine (CML).

Gla	Resonance	δ pH 2 (ppm)	δ pH 9 (ppm)	CML	Resonance	δ pH 2 (ppm)	δ pH 9 (ppm)
	NH	119.0	121.0[a]		NH	120.9	120.9[a]
	HN	8.45	8.65[a]		HN	8.31	8.32[a]
	Hα	4.49	4.27		Hα	4.35	4.35
	Hβ	2.26, 2.48	2.14, 2.27		Hβ	1.78, 1.88	1.78, 1.88
	Hγ	3.54	3.15		Hγ	1.43, 1.47	1.42, 1.46
					Hδ	1.73	1.71
					Hε	3.09	3.02
					H_2C	3.81	3.57
	Cα	54.7	56.4		Cα	56.4	56.5
	Cβ	32.6	34.2		Cβ	32.9	32.9
	Cγ	51.9	57.9		Cγ	24.8	24.8
	Cδ	175.7	180.9		Cδ	27.7	27.9
					Cε	50.0	50.0
					CH_2	50.9	52.2
					C=O(CML)	172.8	174.8
	C=O	176.3	177.8		C=O	177.5	177.6

[a] Determined at pH 6.5 as signals are not visible at pH 9 due to rapid exchange with the solvent.

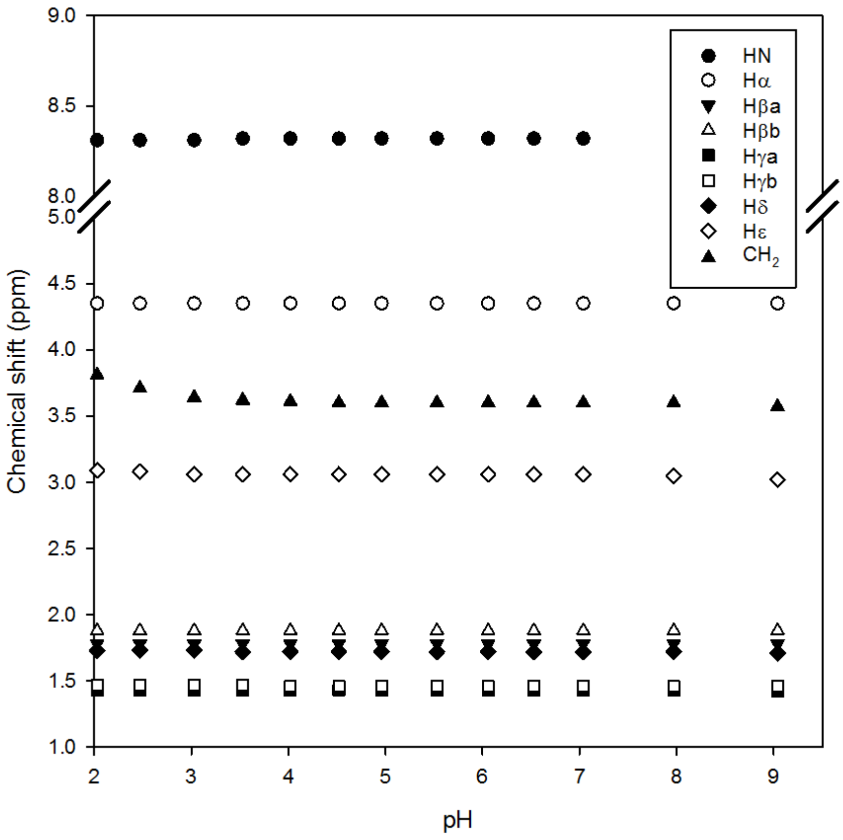

Figure S1. pH dependence of ^{1}H chemical shifts in carboxymethyl lysine (CML).

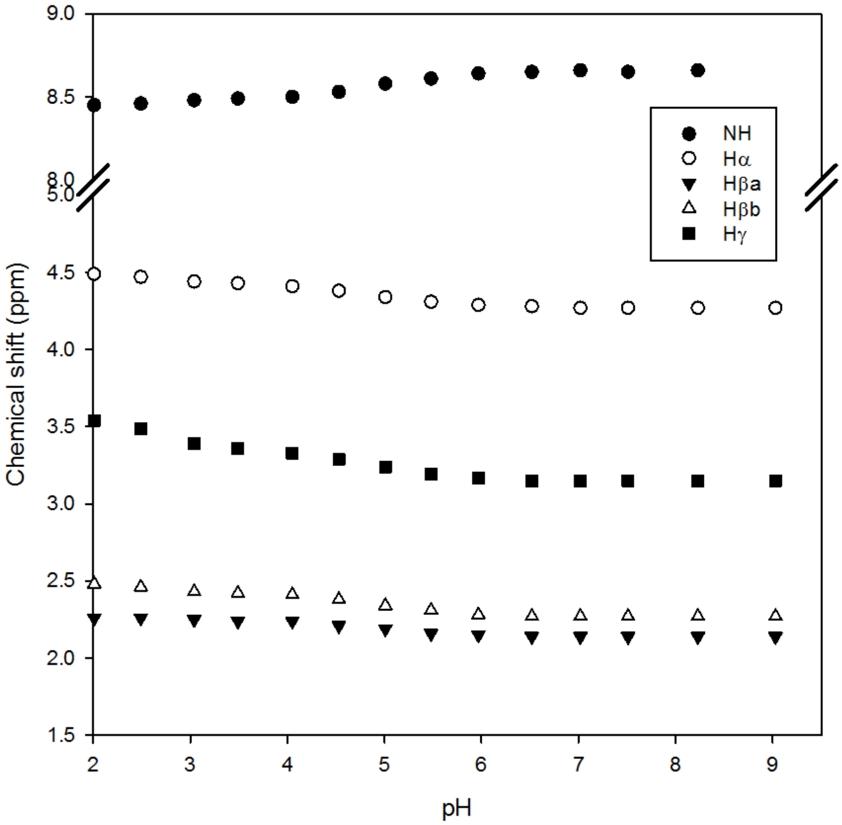

Figure S2. pH dependence of ^{1}H chemical shifts in γ-carboxy glutamic acid (Gla).

Structures and mass spectra of random coil peptides containing modified amino acids.

The mass spectra shown are for the crude peptides after lyophilisation. We have attempted to identify significant peaks that appear in addition to the mass of the desired peptide, most of which are likely to be artefacts of the ionization process in the mass spectrometer. The most common of these are a peak corresponding to the dimer 2(M+H)$^+$, addition or loss of water or –NH$_2$ (+/- 17) due to cyclisation and addition of sodium (+ 23) or potassium ions (+ 39). We also sometimes observed addition and deletion products of glycine (+/- 57) that occur during SPPS. In all cases, the desired peptide mass was the highest intensity peak and the peptides were of sufficient purity for unambiguous assignment of the resonances from the NMR spectra.

Phosphoserine

Ac-G-G-pS-G-G-NH$_2$,

MW$_{calc}$: 454.3, (M+H)$^+_{obs}$: 455.2

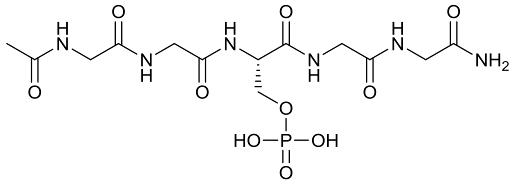

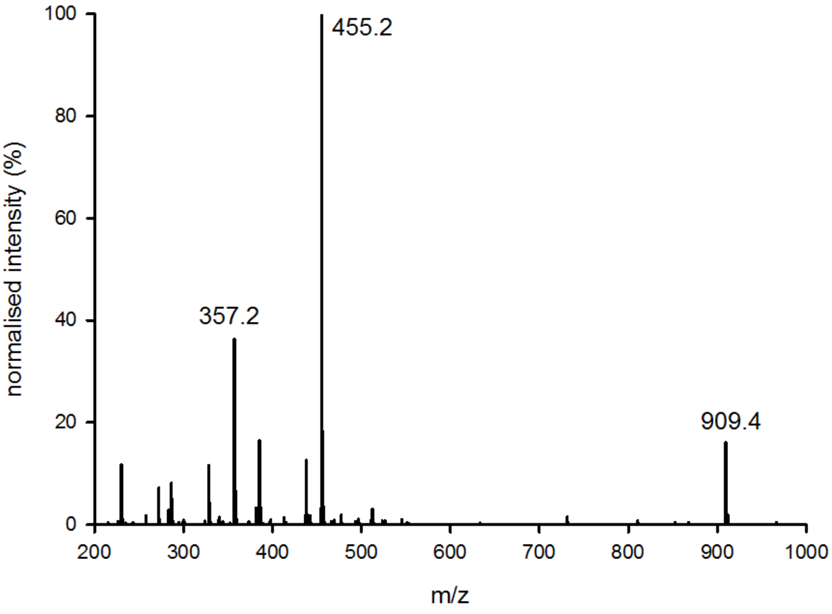

Additional peaks: 909.4 = 2(M+H)$^+$; 357.2 = -98 (elimination of the phosphate group and dehydration/cyclisation)

Phosphothreonine

Ac-G-G-pT-G-G-NH2,

MW_{calc}: 468.4, $(M+H)^{+}_{obs}$: 469.2

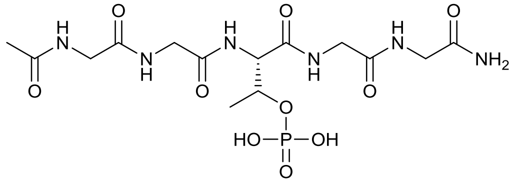

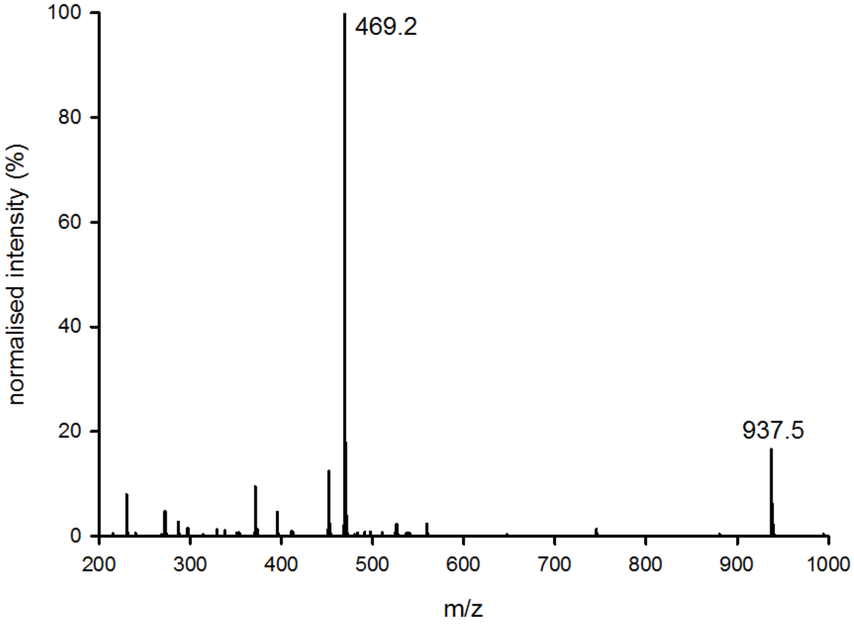

Additional peaks: 937.5 = $2(M+H)^{+}$

Phosphotyrosine

Ac-G-G-pY-G-G-NH_2,

MW_{calc}: 530.4, $(M+H)^+_{obs}$: 531.1

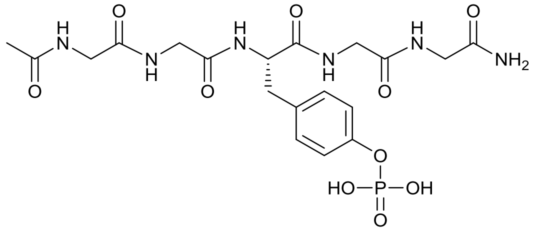

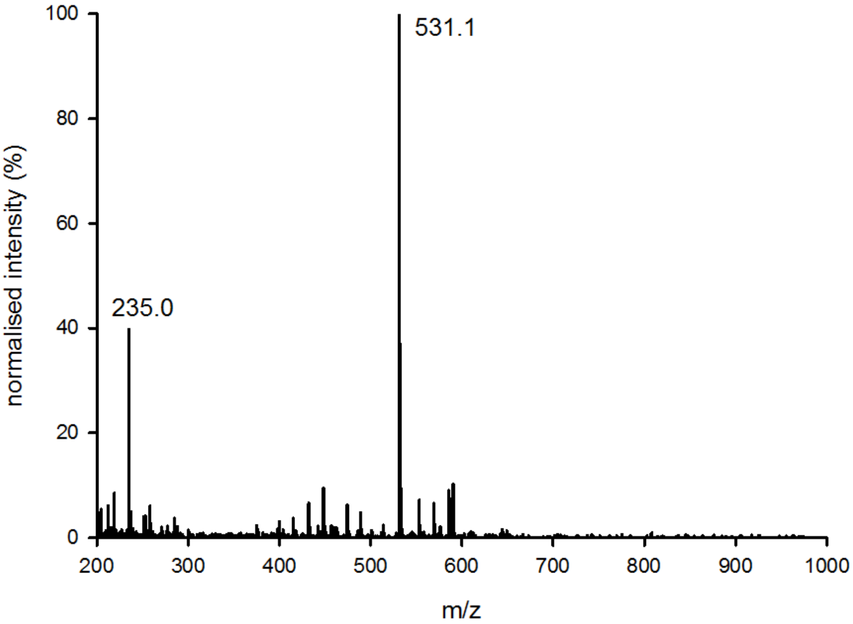

Additional peaks: 235.0 could not be identified.

Acetyllysine

Ac-G-G-K(ac)-G-G-NH_2,

MW_{calc}: 457.5, $(M+H)^+_{obs}$: 458.3

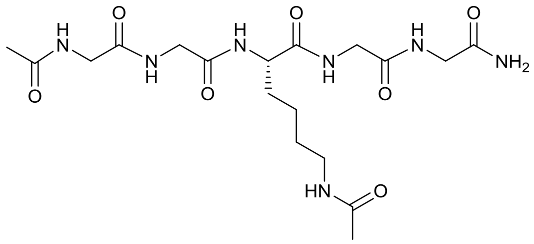

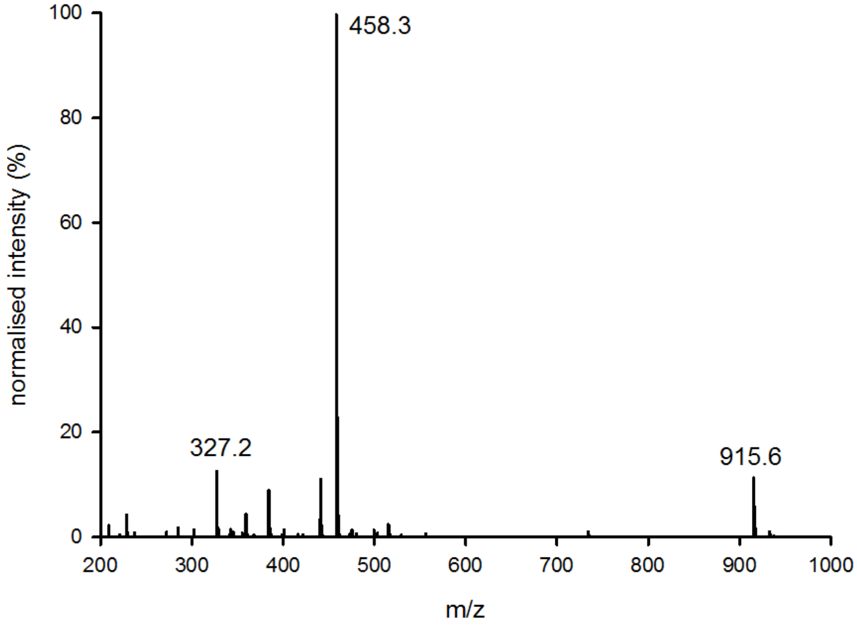

Additional peaks: 915.6 = $2(M+H)^+$; 327.2 could not be identified.

Dimethylarginine (symmetric)

Ac-G-G-(SDMA)-G-G-NH_2,

MW_{calc}: 471.5, $(M+H)^{+}_{obs}$: 472.2

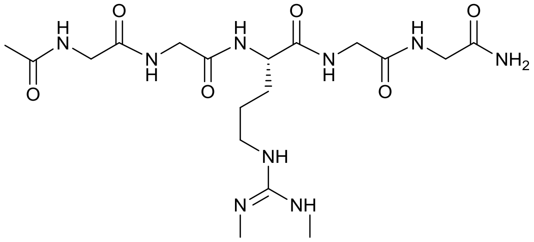

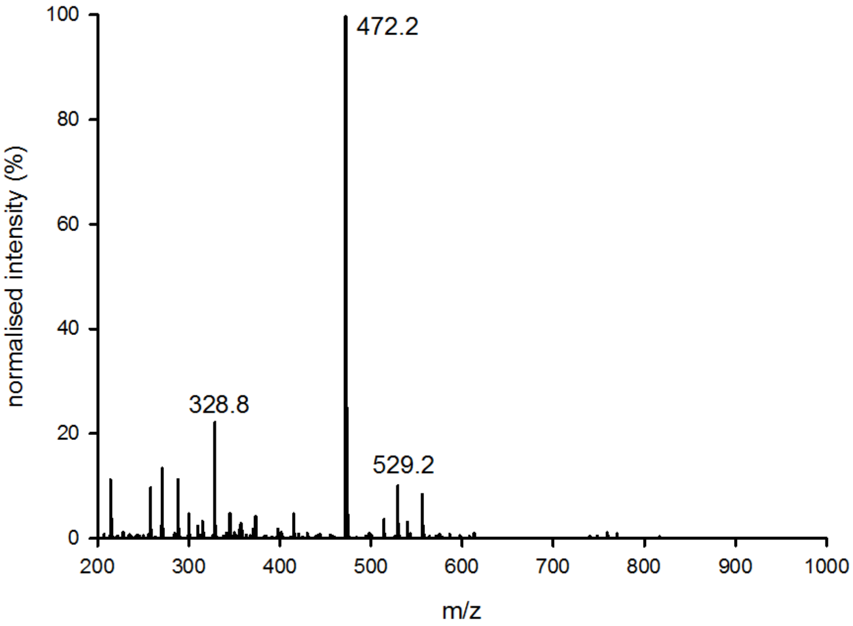

Additional peaks: 328.8 could not be identified; 529.2 = + 57 (glycine addition).

Dimethylarginine (asymmetric)

Ac-G-G-(ADMA)-G-G-NH_2,

MW_{calc}: 471.5, $(M+H)^+_{obs}$: 472.4

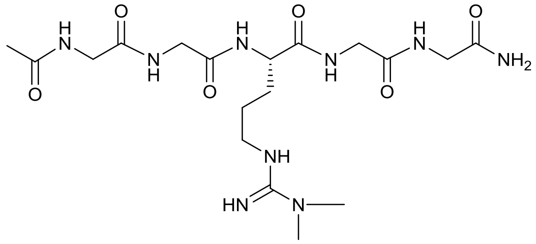

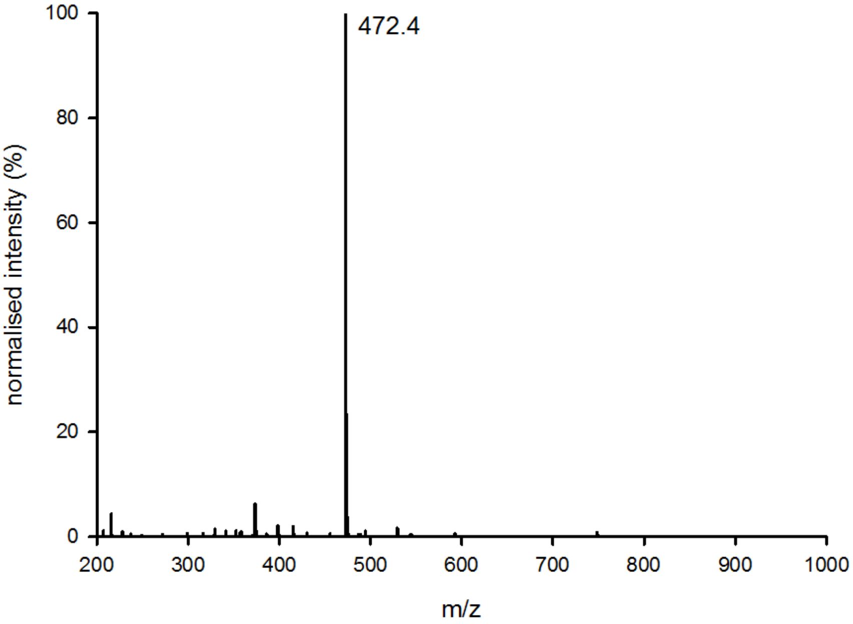

Methylarginine

Ac-G-G-R(Me)-G-G-NH_2,

MW_{calc}: 457.5, $(M+H)^{+}_{obs}$: 458.4

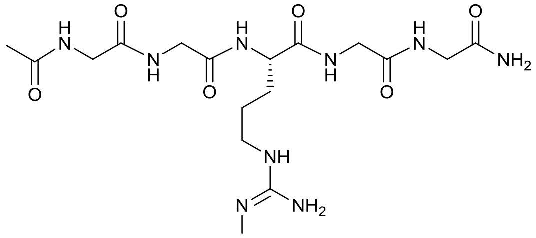

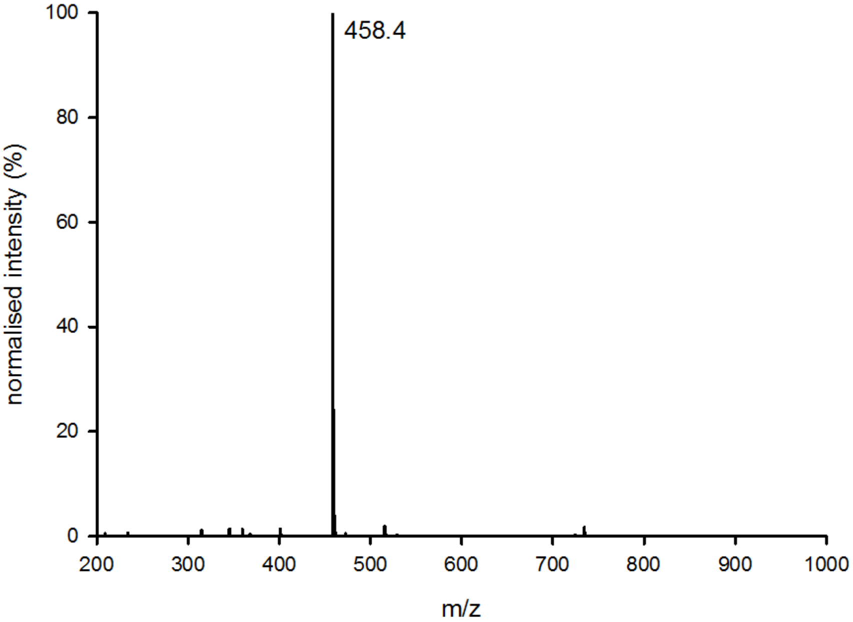

Trimethyllysine

Ac-G-G-K(Me)3-G-G-NH2,

MW_{calc}: 458.5, $(M)^{+}_{obs}$: 458.4

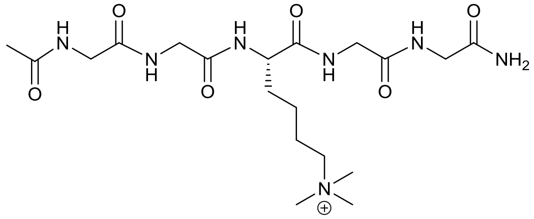

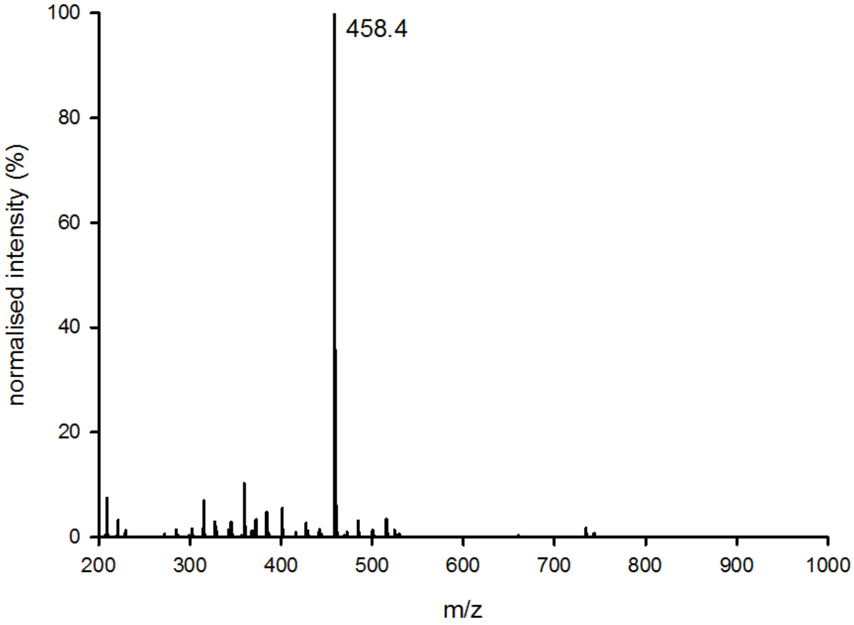

Dimethyllysine

Ac-G-G-K(Me)2-G-G-NH2,

MW_{calc}: 443.5, $(M+H)^{+}_{obs}$: 444.4

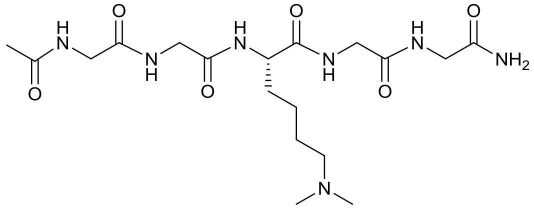

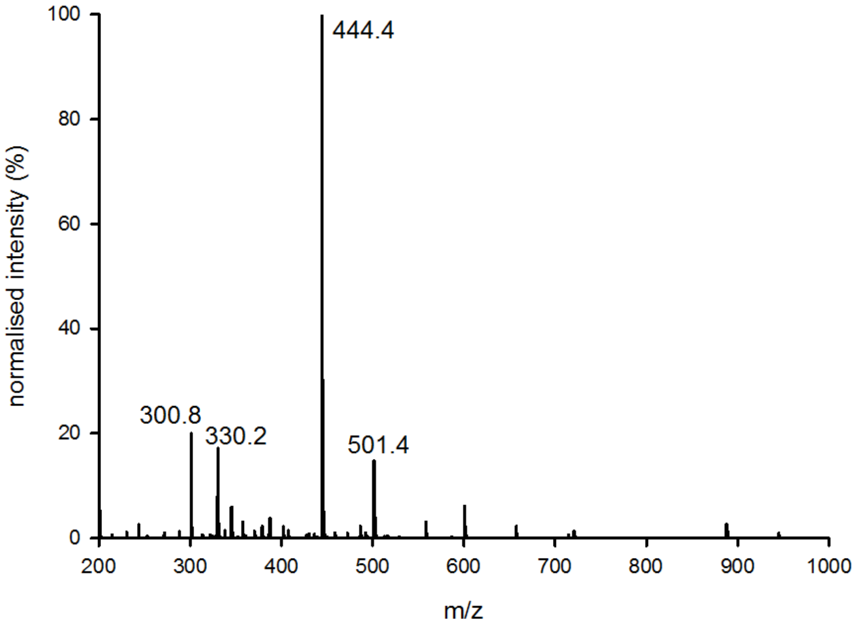

Additional peaks: 501.4 = + 57 (glycine addition); 330.2 = - 114.2 (2 x glycine deletion); 300.8 could not be identified.

Methyllysine

Ac-G-G-K(Me)-G-G-NH_2,

MW_{calc}: 429.5, $(M+H)^{+}_{obs}$: 430.4

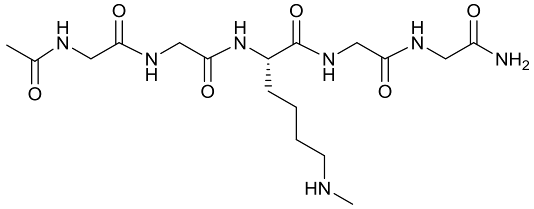

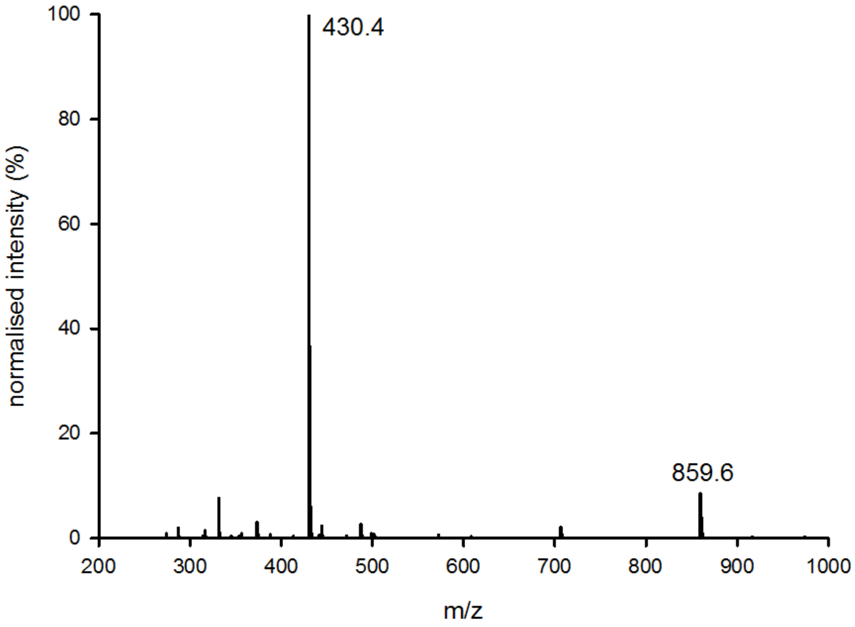

Additional peaks: 859.6 = $2(M+H)^{+}$

Argpyrimidine

Ac-G-G-Apy-G-G-NH_2,

MW_{calc}: 523.6, $(M+H)^{+}_{obs}$: 524.3

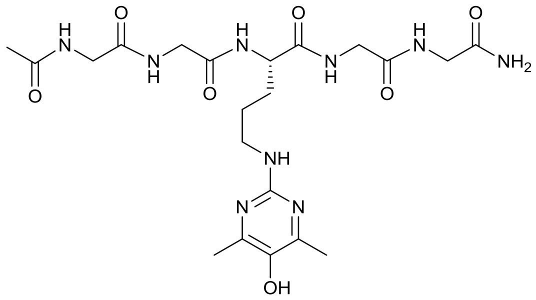

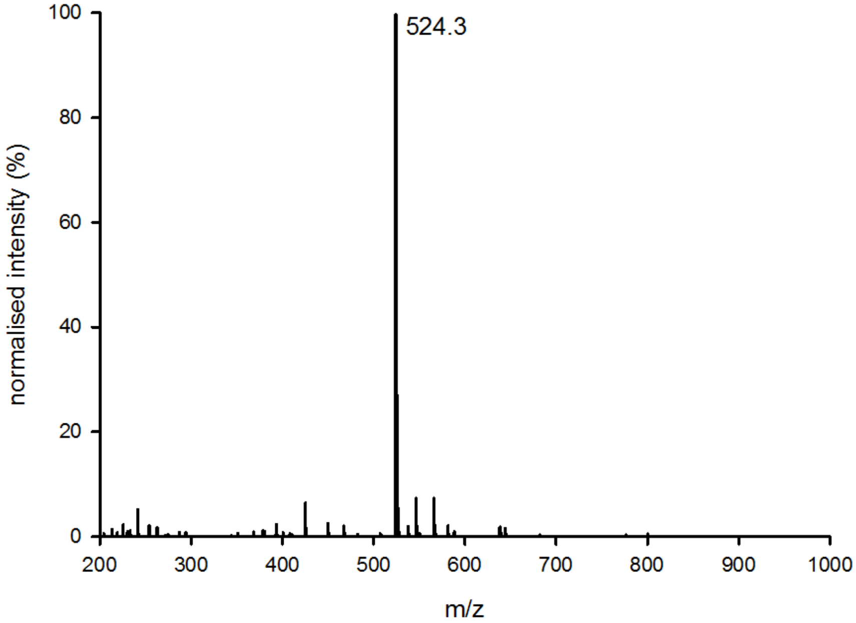

4-hydroxyproline

Ac-G-G-Hyp-G-G-NH_2,

MW_{calc}: 400.4, $(M+H)^+_{obs}$: 401.1

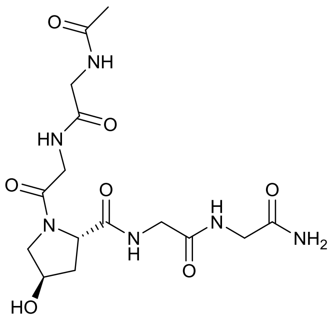

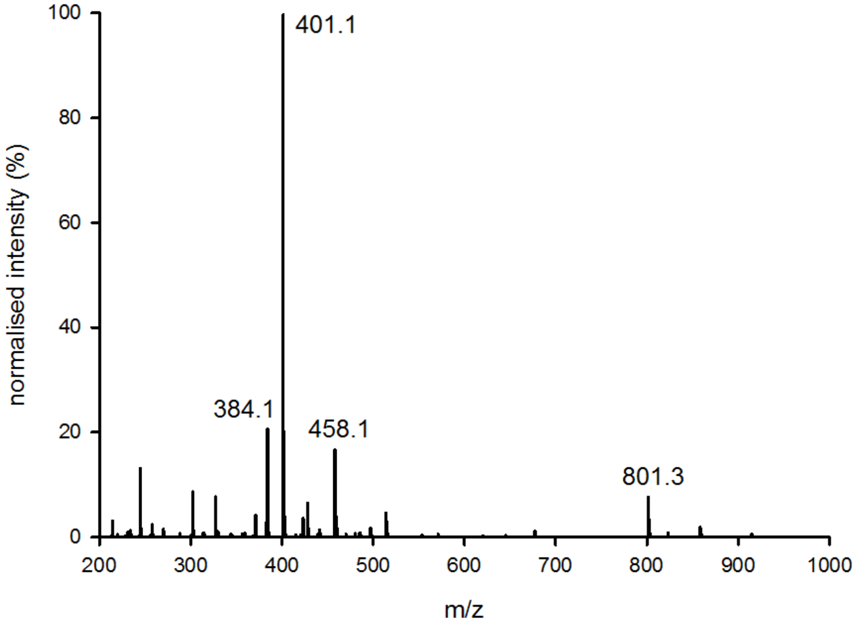

Additional peaks: 801.3 = $2(M+H)^+$; 458.1 = + 57 (glycine addition); 384.1 = - 17 (cyclisation/dehydroxylation?)

Citrulline

Ac-G-G-Cit-G-G-NH_2,

MW_{calc}: 444.5, $(M+H)^+_{obs}$: 445.3

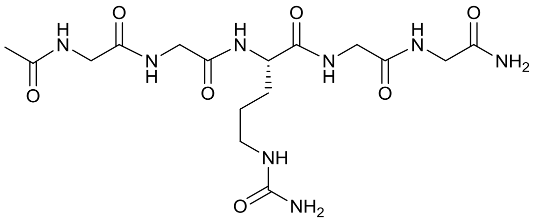

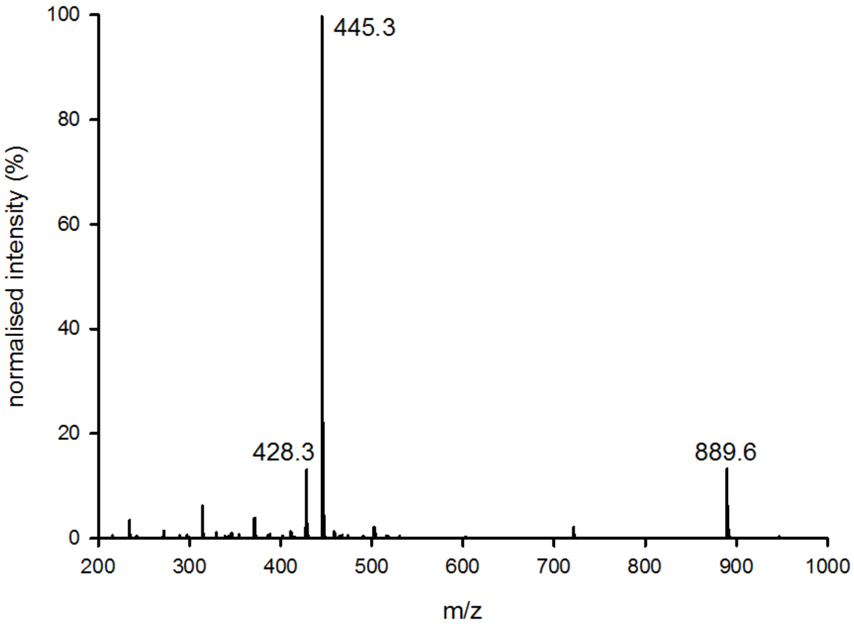

Additional peaks: 889.6 = $2(M+H)^+$; 428.3 = - 17 (dehydroxlyation/deamidation/cyclisation?)

Threonine(GalNAc)

Ac-G-G-Thr(α-D-GalNAc)-G-G-NH_2,

MW_{calc}: 591.6, $(M+H)^+_{obs}$: 592.2

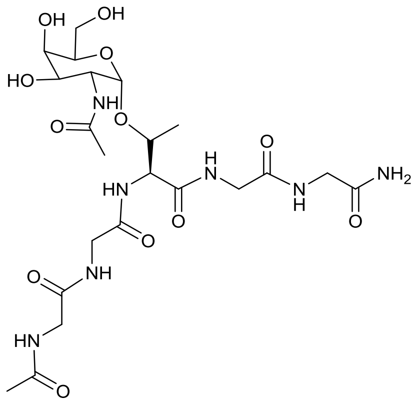

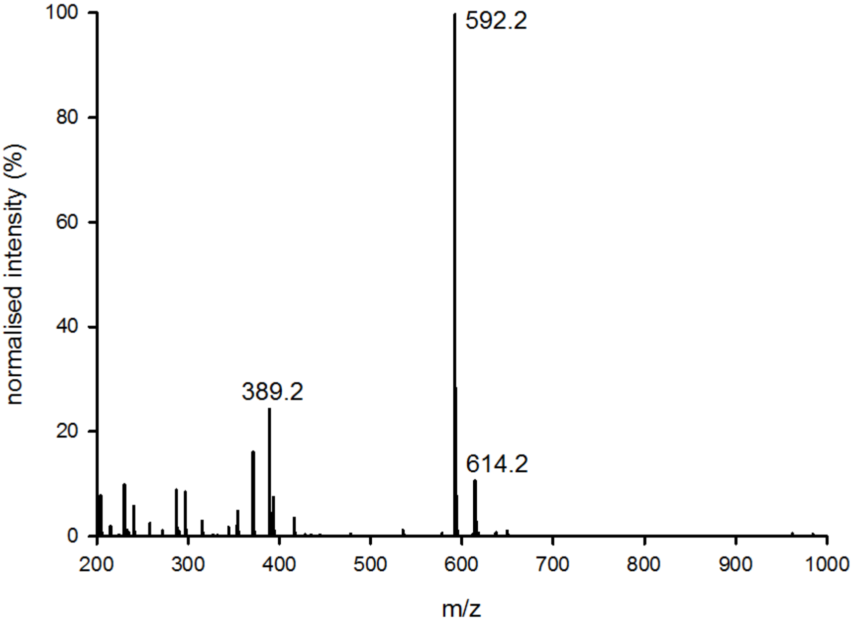

Additional peaks: 614.2 = + 22 (M + Na^+); 389.2 = - 203 (hydrolysis of GalNAc)

Asparagine(GlcNAc)

Ac-G-G-Asn(β-D-GlcNAc)-G-G-NH_2,

MW_{calc}: 604.6, $(M+H)^{+}_{obs}$: 605.2

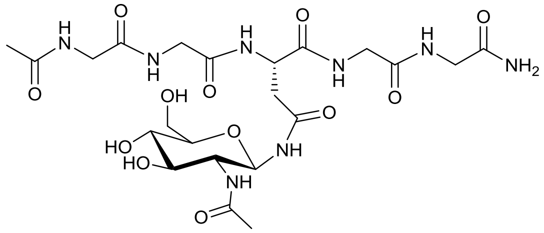

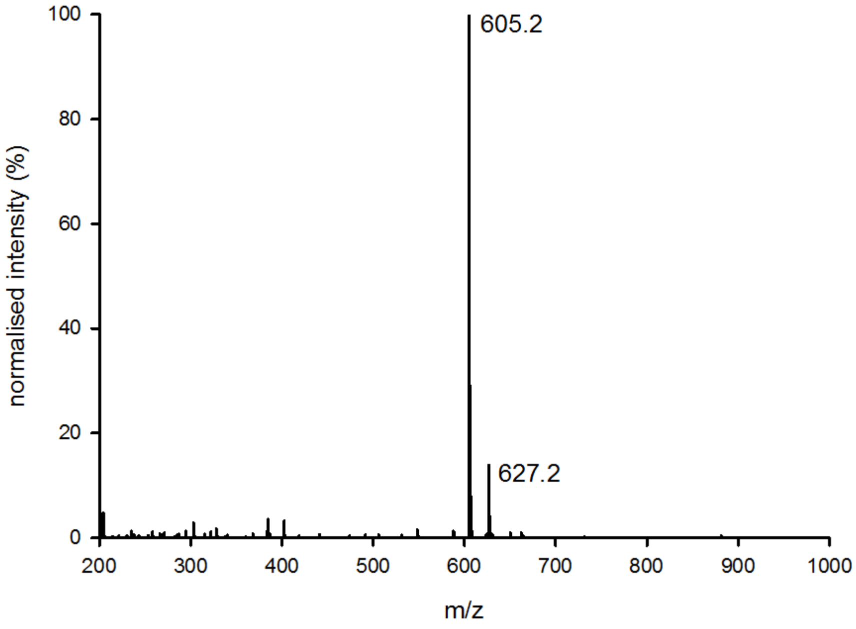

Additional peaks: 627.2 = + 22 ($M + Na^{+}$)

Sulfotyrosine

Ac-G-G-Tyr(SO_3)-G-G-NH_2,

MW_{calc}: 530.5, $(M\text{-}H)^-_{obs}$: 529.0

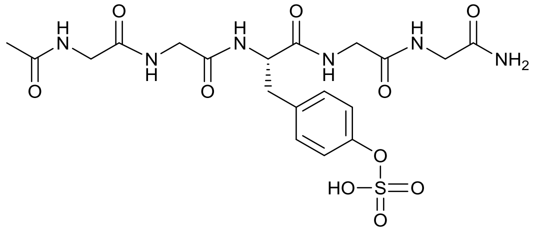

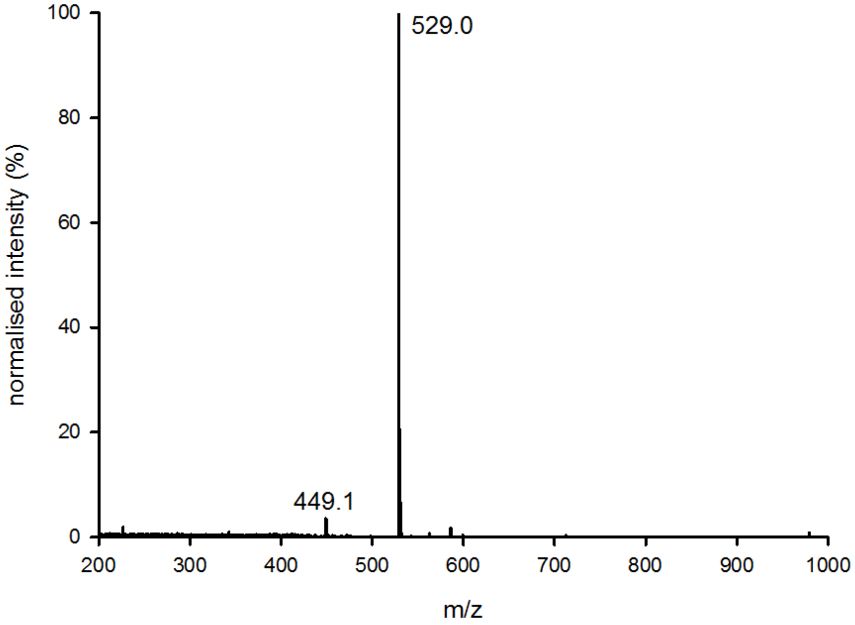

Additional peaks: 449.1 = - 80 (desulfurization)

γ-carboxyglutamate

Ac-G-G-Gla-G-G-NH_2,

MW_{calc}: 460.4, $(M+H)^+_{obs}$: 461.3

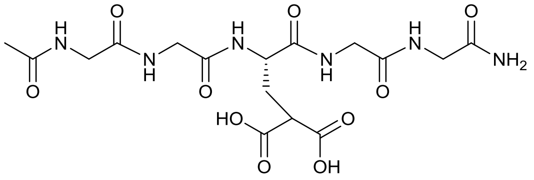

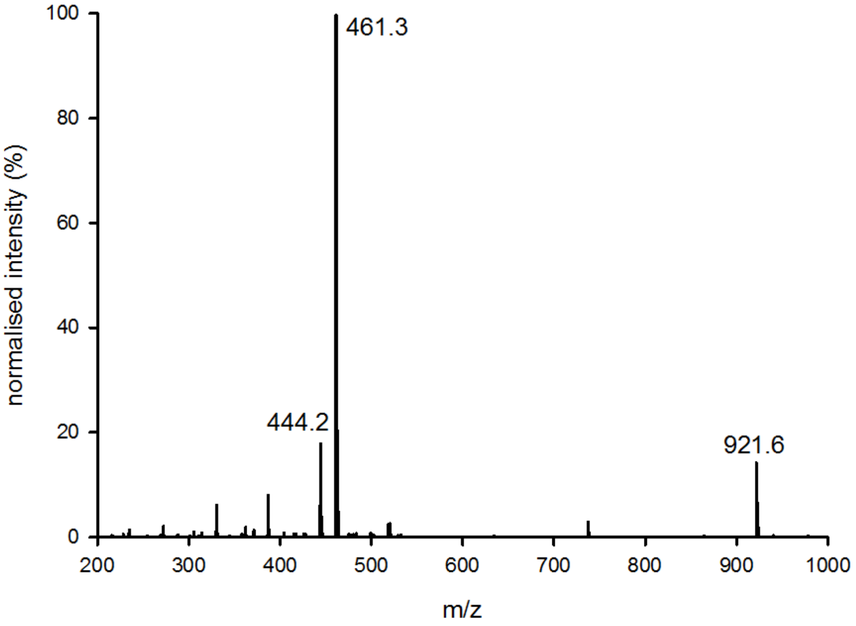

Additional peaks: 921.6 = $2(M+H)^+$; 444.2 = - 17 (dehydroxylation/cyclisation?)

Carboxymethyllysine

Ac-G-G-CML-G-G-NH_2,

MW_{calc}: 473.5, $(M+H)^+_{obs}$: 474.3

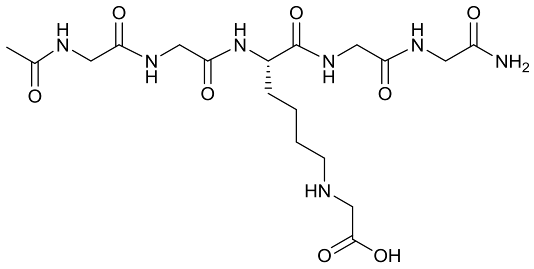

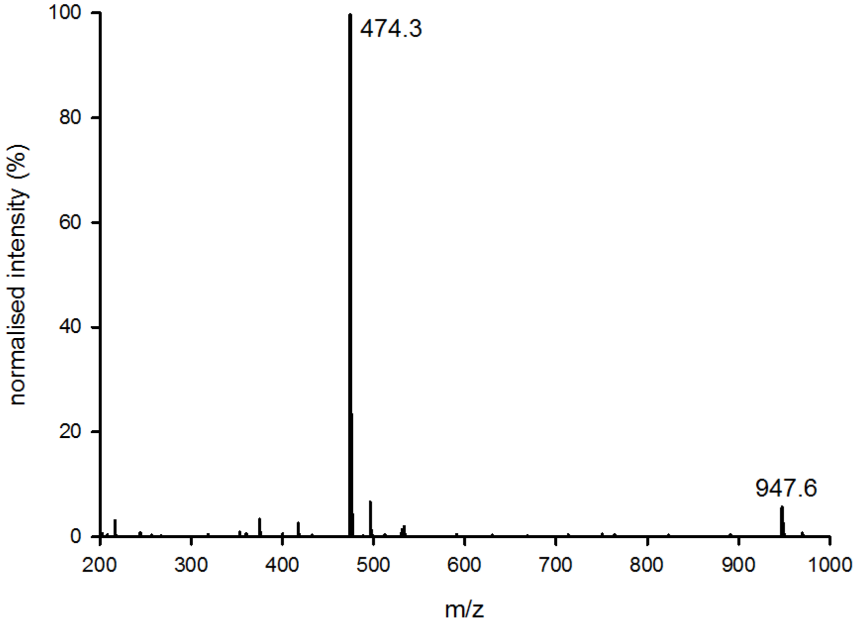

Additional peaks: 947.6 = $2(M+H)^+$

Structures and mass spectra of random coil peptides containing standard amino acids.

Serine

Ac-G-G-S-G-G-NH_2,

MW_{calc}: 374.4, $(M+H)^{+}_{obs}$: 375.2

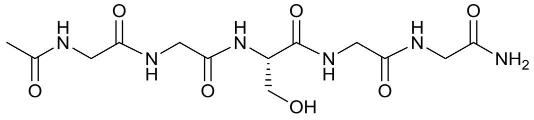

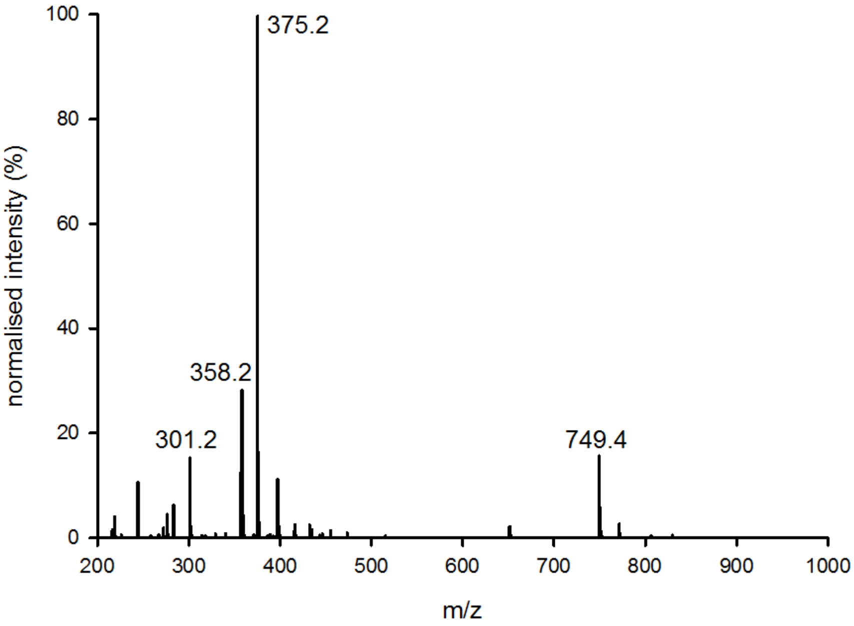

Additional peaks: 749.4 = $2(M+H)^+$; 358.2 = -17 (β-elimination with loss of water?); 301.2 = -74 (glycine deletion – water?).

Threonine

Ac-G-G-T-G-G-NH_2,

MW_{calc}: 388.4, $(M+H)^+_{obs}$: 389.2

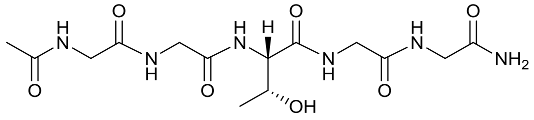

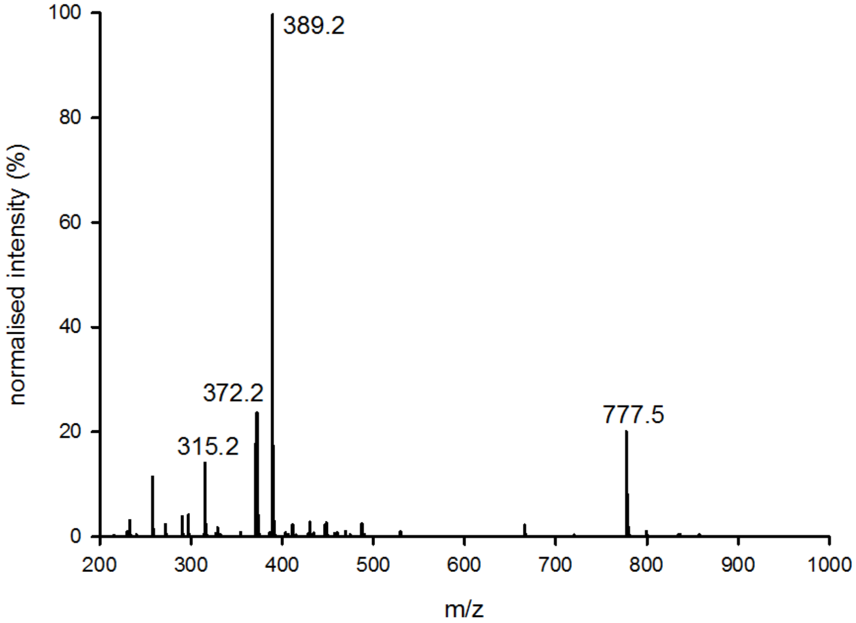

Additional peaks: 777.5 = $2(M+H)^+$; 372.2 = -17 (β-elimination with loss of water?); 315.2 = -74 (glycine deletion – water?).

Tyrosine

Ac-G-G-Y-G-G-NH_2,

MW_{calc}: 450.5, $(M+H)^+_{obs}$: 451.2

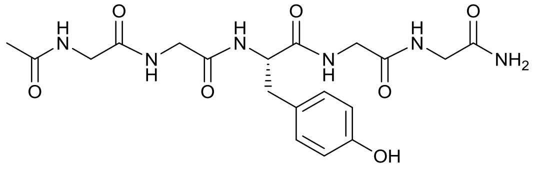

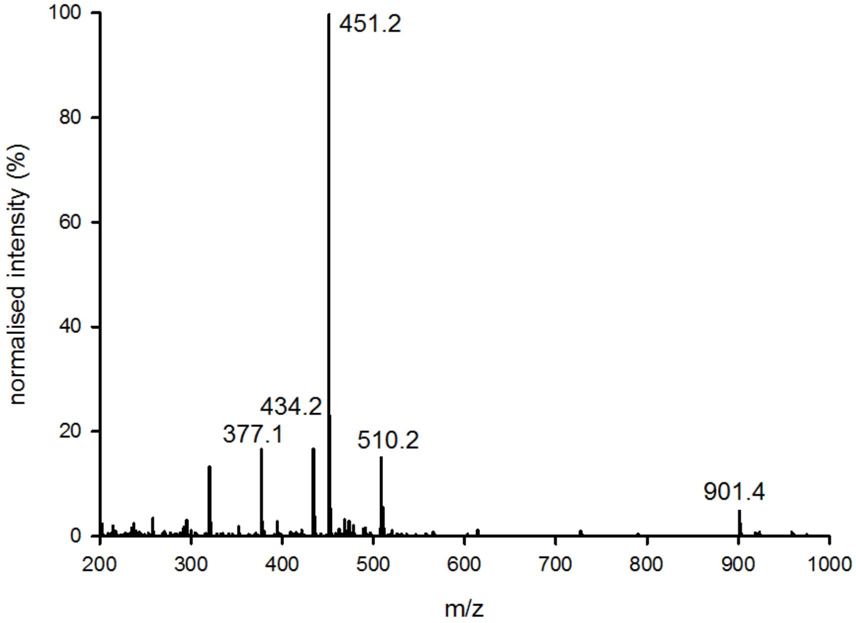

Additional peaks: 901.4 = $2(M+H)^+$; 510.2 = + 17 (addition of water?); 434.2 = -17 (loss of water?); 377.1 = -74 (glycine deletion – water?).

Lysine

Ac-G-G-K-G-G-NH_2,

MW_{calc}: 415.5, $(M+H)^+_{obs}$: 416.2

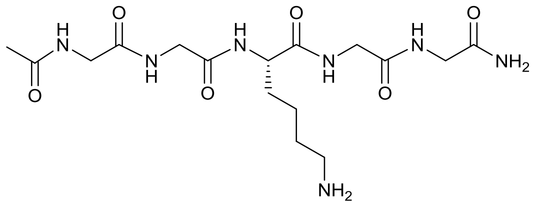

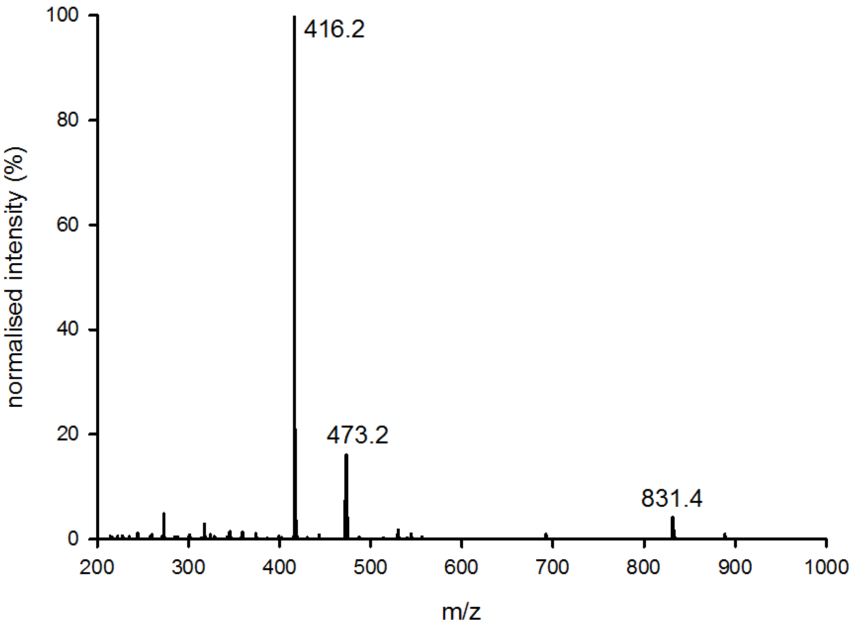

Additional peaks: 831.4 = $2(M+H)^+$; 473.2 = + 57 (glycine addition).

Arginine

Ac-G-G-R-G-G-NH_2,

MW_{calc}: 443.5, $(M+H)^+_{obs}$: 444.2

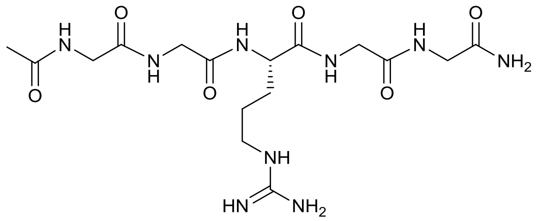

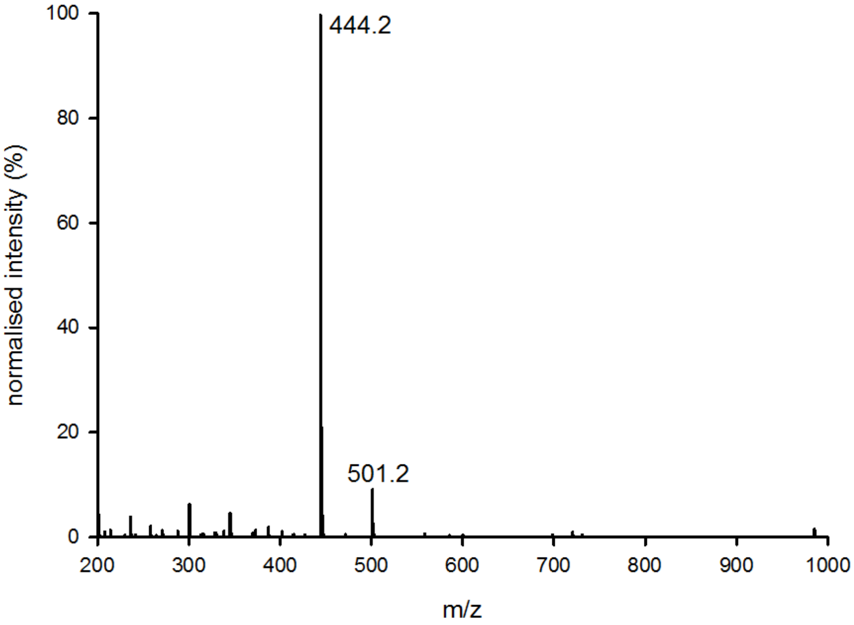

Additional peaks: 501.2 = + 57 (glycine addition).

Proline

Ac-G-G-P-G-G-NH_2,

MW_{calc}: 384.4, $(M+H)^+_{obs}$: 385.1

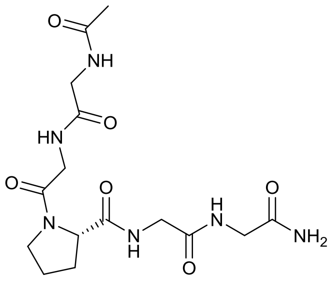

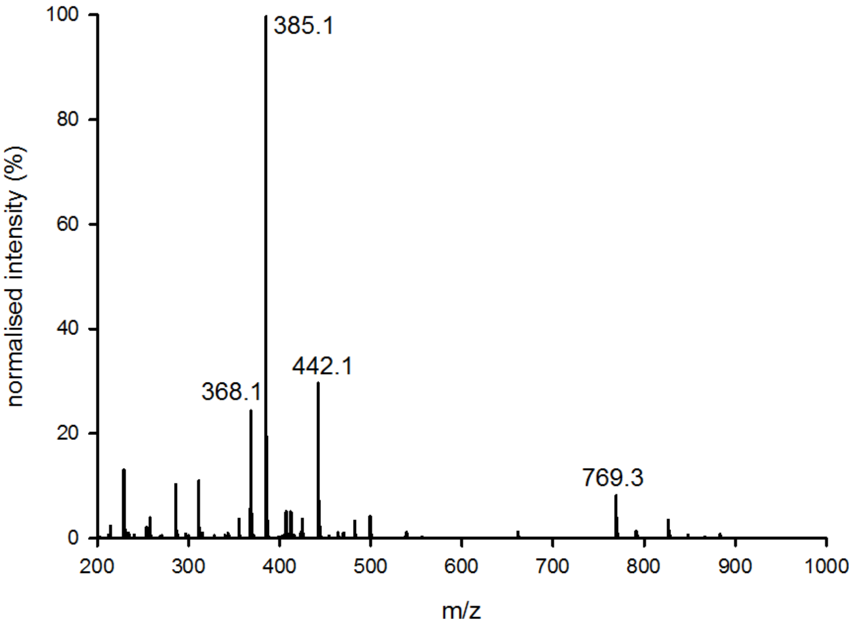

Additional peaks: 769.3 = $2(M+H)^+$; 442.1 = + 57 (glycine addition); 368.1 = - 17 (loss of water/cyclisation?).

Asparagine

Ac-G-G-N-G-G-NH_2,

MW_{calc}: 401.4, $(M+H)^{+}_{obs}$: 402.2

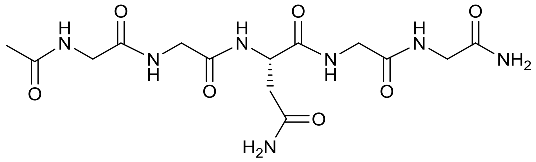

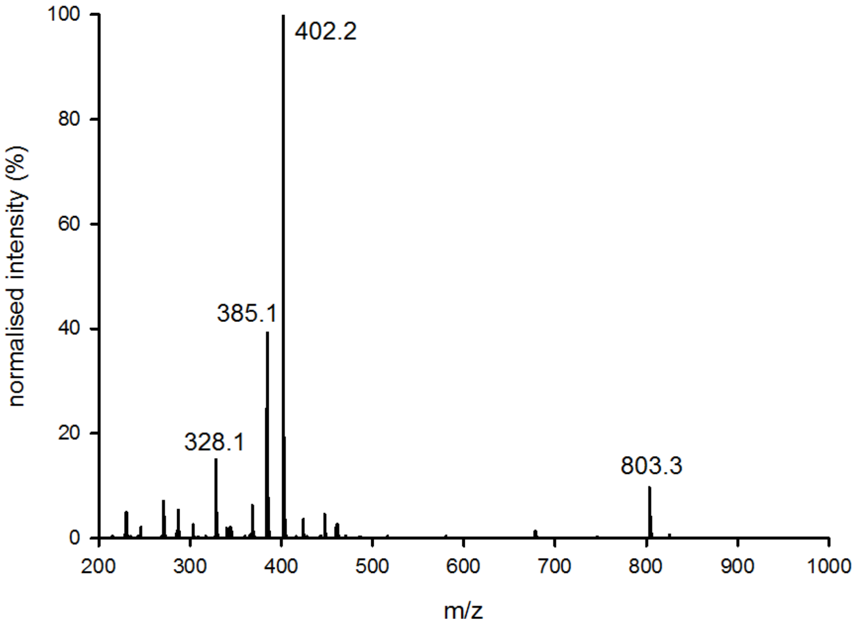

Additional peaks: 803.3 = $2(M+H)^{+}$; 385.1 = - 17 (loss of water/amine?); - 74 (glycine deletion and loss of water/amine?).

Glutamic acid

Ac-G-G-E-G-G-NH_2,

MW_{calc}: 416.4, $(M+H)^{+}_{obs}$: 417.3

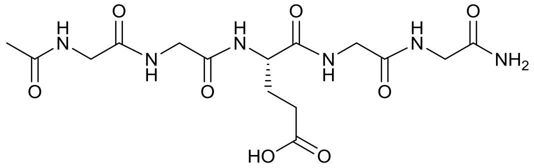

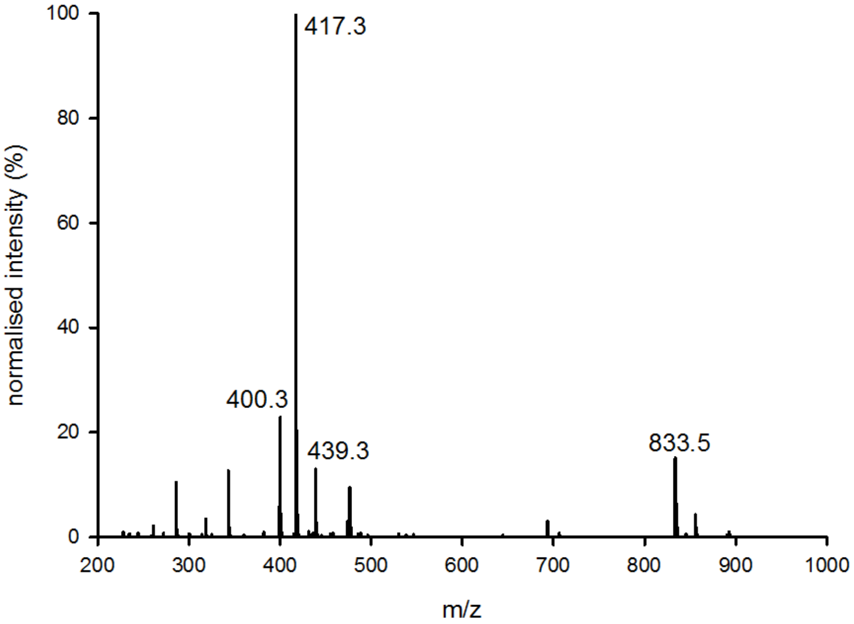

Additional peaks: 833.5 = $2(M+H)^{+}$; 439.3 = +22 (addition of Na^{+}?); 400.3 = - 17 (loss of water/cyclisation?).

Glycine

Ac-G-G-G-G-G-NH_2,

MW_{calc}: 344.3, $(M+H)^{+}_{obs}$: 345.2

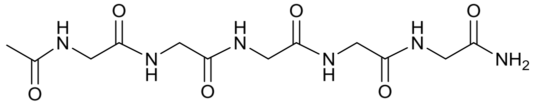

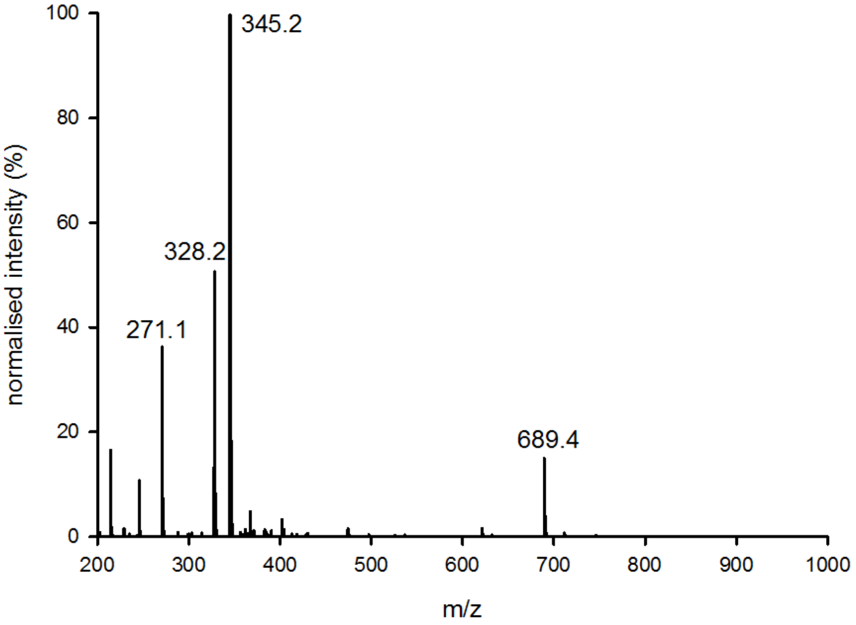

Additional peaks: 689.4 = $2(M+H)^{+}$; 328.2 = - 17 (loss of water?); 271.1 = -74 (glycine deletion and loss of water/cyclisation).
